# Supplementary material for: Fenretinide inhibits obesity and fatty liver disease but induces Smpd3 to increase serum ceramides and worsen atherosclerosis in LDLR−/− mice
Source: Sci Rep. 2023 Mar 9;13:3937. doi: 10.1038/s41598-023-30759-w (PMC9998859; doi:10.1038/s41598-023-30759-w)

**This Supplementary Information accompanies the paper**

**Fenretinide inhibits obesity and fatty liver disease but induces *Smpd3* to increase serum ceramides and worsen atherosclerosis in LDLR-/- mice**

Dawn Thompson et al.,…. & Nimesh Mody

**Supplemental Material and Methods**

**Animal studies.** All animal procedures were performed under a project licence (PPL P94B395E0) approved by the U.K. Home Office under the Animals (Scientific Procedures) Act 1986 and the University of Aberdeen ethics review board. Studies were performed following the recommendations in the ARRIVE guidelines under guidance by the Veterinary Surgeon and Animal Care and Welfare Officers of the institutional animal research facility. Thus, all methods were performed in accordance with the relevant guidelines and regulations.

**Immunoblotting.**

Tissues were prepared as previously described in materials and methods. Blots used in figures are all compliant with the digital image and integrity policies of Nature publishing and Scientific Reports journal. Western blot membranes were cut at approximate molecular weight ( +/- 20 kDa ) of target protein before incubation of primary antibodies. Equal numbers of representative samples from all treatment groups were run on multiple gels/blots to accommodate all samples. Images obtained were minimally processed. Image analysis and quantification with normalisation to loading control protein was performed within the same membrane and then data combined for graphical representation. No direct quantitative comparisons between samples on different gels/blots were performed.

Membranes were probed for the following additional proteins;

Cell Signaling Technology

phospho-p38 (The 181/Tyr 182, cat: 4581),

total p-38 (cat: 8690), phospho-eif2α (Ser 51, cat: 3398),

total eif2α (cat: 5321),

beclin 1 (cat: 3495) and

GAPDH (cat: 5174,),

or Santa Cruz Biotechnology

IR β-chain (cat: sc-373975),

Bip (sc166490) and

CHOP (sc-7531),

and

RBP4 (Dako)

**Supplemental Tables and Figures**

**Supplemental Table 1:** qPCR primer details.

| Gene | Forward primer (5’-3’) | Reverse Primer (5’-3’) |
| --- | --- | --- |
| *Abca1* | AAAACCGCAGACATCCTTCAG | CATACCGAAACTCGTTCACCC |
| *Abcc3* | CTGGGTCCCCTGCATCTAC | GCCGTCTTGAGCCTGGATAAC |
| *Abcg1* | GTGGATGAGGTTGAGACAGACC | CCTCGGGTACAGAGTAGGAAAG |
| *Acadm* | AGGGTTTAGTTTTGAGTTGACGG | CCCCGCTTTTGTCATATTCCG |
| *Acox1* | TAACTTCCTCACTCGAAGCCA | AGTTCCATGACCCATCTCTGTC |
| *Adiponectin* | TGTTCCTCTTAATCCTGCCCA | CCAACCTGCACAAGTTCCCTT |
| *Apoa1* | GGCACGTATGGCAGCAAGAT | CCAAGGAGGAGGATTCAAACTG |
| *Apoa2* | GCAGACGGACCGGATATGC | GCTGCTCGTGTGTCTTCTCA |
| *Apoc3* | TACAGGGCTACATGGAACAAGC | CAGGGATCTGAAGTGATTGTCC |
| *Apoe* | CTGACAGGATGCCTAGCCG | CGCAGGTAATCCCAGAAGC |
| *Cd36* | GAACCACTGCTTTCAAAAACTG G | TGCTGTTCTTTGCCACGTCA |
| *Cd68* | TGTCTGATCTTGCTAGGACCG | GAGAGTAACGGCCTTTTTGTGA |
| *Cers2* | AAGTGGGAAACGGAGTAGCG | ACAGGCAGCCATAGTCGTTC |
| *Cers6* | CGGCTGGGCATATTTCCTCT | GTCATCCTTGGATACCTTGCCT |
| *Col1a1* | CCAAAGGTGCTGATGGTTCT | ACCAGCTTCACCCTTGTCAC |
| *Col4a1* | GCTCTGGCTGTGGAAAATGT | CTTGCATCCCGGGAAATC |
| *Cpt1a* | CTCCGCCTGAGCCATGAAG | CACCAGTGATGATGCCATTCT |
| *Cyp26A1* | TTCGGGTTGCTCTGAAGACT | TCCTCCAAATGGAATGAAGC |
| *Degs1* | TCCCTACTCGCGGATGAAGA | TTTGAAGCCGTGGACAGGAA |
| *Dgat* | TCCGTCCAGGGTGGTAGTG | TGAACAAAGAATCTTGCAGACGA |
| *G6Pase* | ATGAACATTCTCCATGACTTTGGG | GACAGGGAACTGCTTTATTATAGG |
| *Glut4* | GGAAGGAAAAGGGCTATGCTG | TGAGGAACCGTCCAAGAATGA |
| *Hmgcr* | GATTCTGGCAGTCAGTGGGAA | GTTGTAGCCGCCTATGCTCC |
| *Hsd17b13* | ATTCCCCGGAGAAGGAAATCT | CAGCCTGCCTATTCCGTGT |
| *Il-10* | GCTCTTACTGACTGGCATGAG | CGCAGCTCTAGGAGCATGTG |
| *Il1β* | GCAACTGTTCCTGAACTCAACT | ATCTTTTGGGGTCCGTCAACT |
| *Il-6* | TAGTCCTTCCTACCCCAATTTCC | TTGGTCCTTAGCCACTCCTTC |
| *Lrat* | CCGTCCCTATGAAATCAGCTC | ATGGGCGACACGGTTTTCC |
| *Lxrα* | CTCAATGCCTGATGTTTCTCCT | TCCAACCCTATCCCTAAAGCAA |
| *Lxrβ* | GCCTGGGAATGGTTCTCCTC | AGATGACCACGATGTAGGCAG |
| *Mcp-1* | TTAAAAACCTGGATCGGAACCAA | GCATTAGCTTCAGATTTACGGGT |
| *Mmp2* | CAAGTTCCCCGGCCATGTC | TTCTTGGTCAAGCTCACCTGTC |
| *Mmp9* | GCGTCGTGATCCCCACTTAC | CAGGCCGAATAGGAGCGTC |
| *Mogat1* | TGGACGCCAGTTTGGTTCCAG | TGCTCTGAGGTCGGGTTCA |
| *Nono* | GCCAGAATGAAGGCTTGACTAT | TATCAGGGGGAAGATTGCCCA |
| *Pepck* | GAGATAGCGGCACAAT | TTCAGAGACTATGCGGTG |
| *Pnpla3* | TCACCTTCGTGTGCAGTCTC | CCTGGAGCCCGTCTCTGAT |
| *Pparα* | ACGATGCTGTCCTCCTTGATG | GTGTGATAAAGCCATTGCCGT |
| *Pparγ* | AGTGGAGACCGCCCAGG | GCAGCAGGTTGTCTTGGATGT |
| *Rarα* | CGC CAA GGG AGC TGA ACG GG | GGG TGG CTG GGC TGC TTC TG |
| *Rarβ* | CGCGAGCCCTTCCTCCTGC | AAAAGCCCTTGCACCCCTCGC |
| *Resistin* | AAGAACCTTTCATTTCCCCTCCT | GTCCAGCAATTTAAGCCAATGTT |
| *Rpb4* | ACGAGTCCGTCTTCTGAGCAACTG | GCACAGCTCCTCCTGCCGTT |
| *Rxrα* | ATGGACACCAAACATTTCCTGC | CCAGTGGAGAGCCGATTCC |
| *Rxrβ* | CCACCTCTTACCCCTTCAGC | TGGAAGAACTGATGACTGGGA |
| *Smpd1* | TGGGACTCCTTTGGATGGG | CGGCGCTATGGCACTGAAT |
| *Smpd3* | ACACGACCCCCTTTCCTAATA | GGCGCTTCTCATAGGTGGTG |
| *Srebp1c* | GATGTGCGAACTGGACACAG | CATAGGGGGGGTCAAACAG |
| *Tgf1β* | AGCCCGAAGCGGACTACTAT | CTGTGTGAGATGTCTTTGGTTTTC |
| *Timp1* | GCAACTCGGACCTGGTCATAA | CGGCCCGTGATGAGAAACT |
| *Timp2* | TCAGAGCCAAAGCAGTGAGC | GCCGTGTAGATAAACTCGATGTC |
| *Tm6sf2* | AGTTTCGGCGTTCTCACAGC | GCATAGAGAGGGTCGTAGGTG |
| *Tnfα* | CCCTCACACTCAGATCATCTTCT | GCTACGACGTGGGCTACAG |
| *Vldlr* | GGCAGCAGGCAATGCAATG | GGGCTCGTCACTCCAGTCT |
| *Ywaz* | GAAAAGTTCTTGATCCCCAATG C | TGTGACTGGTCCACAATTCCTT |

**
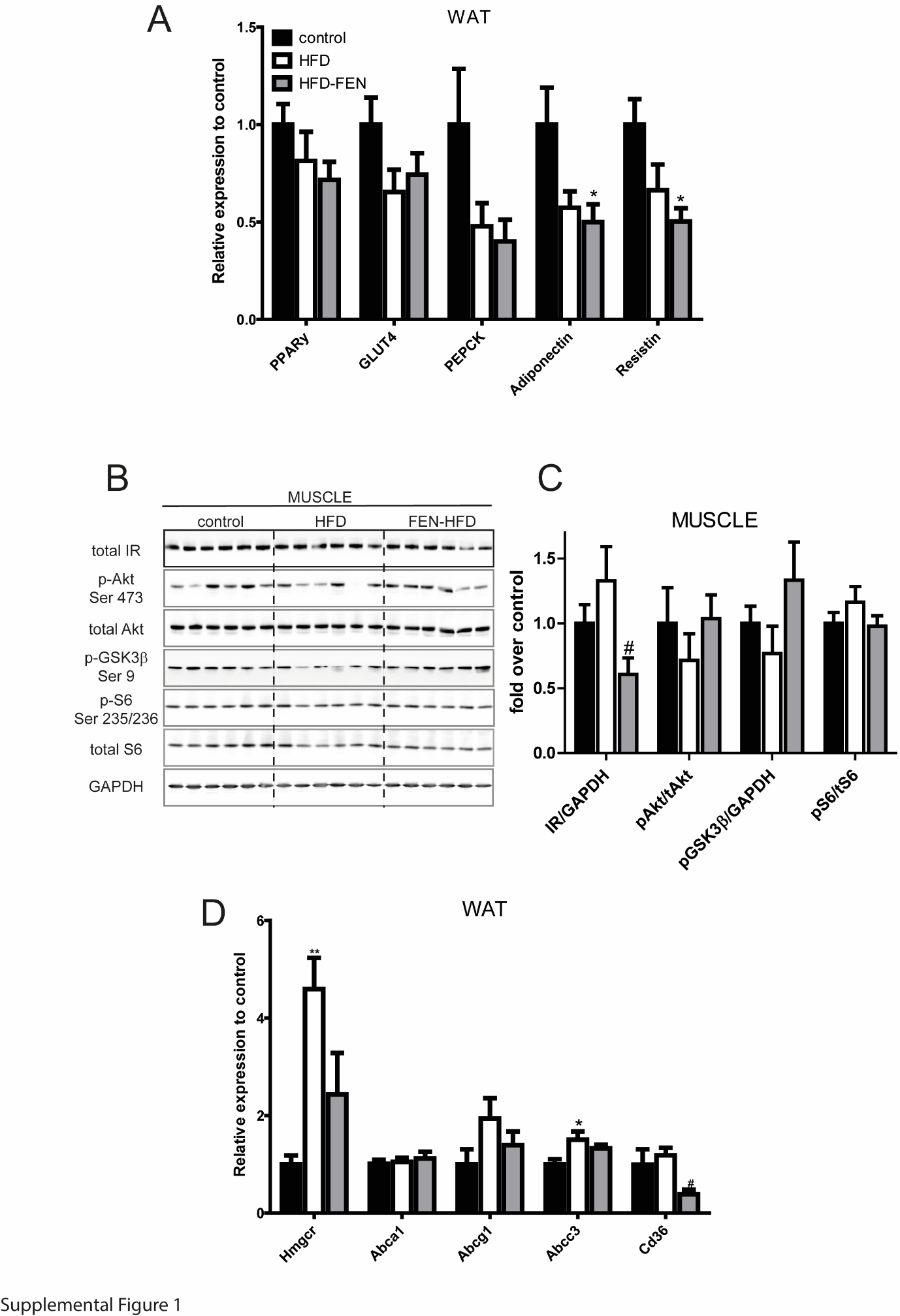
**

**Supplemental Figure 1: WAT and skeletal muscle analysis in LDLR^-/-^ mice.** Genetic analysis of (**A, D**) WAT in LDLR^-/-^ mice (n=8 per group) as analysed by qPCR using SYBR green and LightCycler 480 (Roche). (B) Western blot of skeletal muscle from LDLR-/- mice and quantification shown in (**C**). Data are represented as mean + S.E.M. and analysed by one-way ANOVA followed by Bonferroni multiple comparison t-tests where *p≤0.05 (control compared to HFD) or #p≤0.05 (HFD compared to FEN-HFD).

**
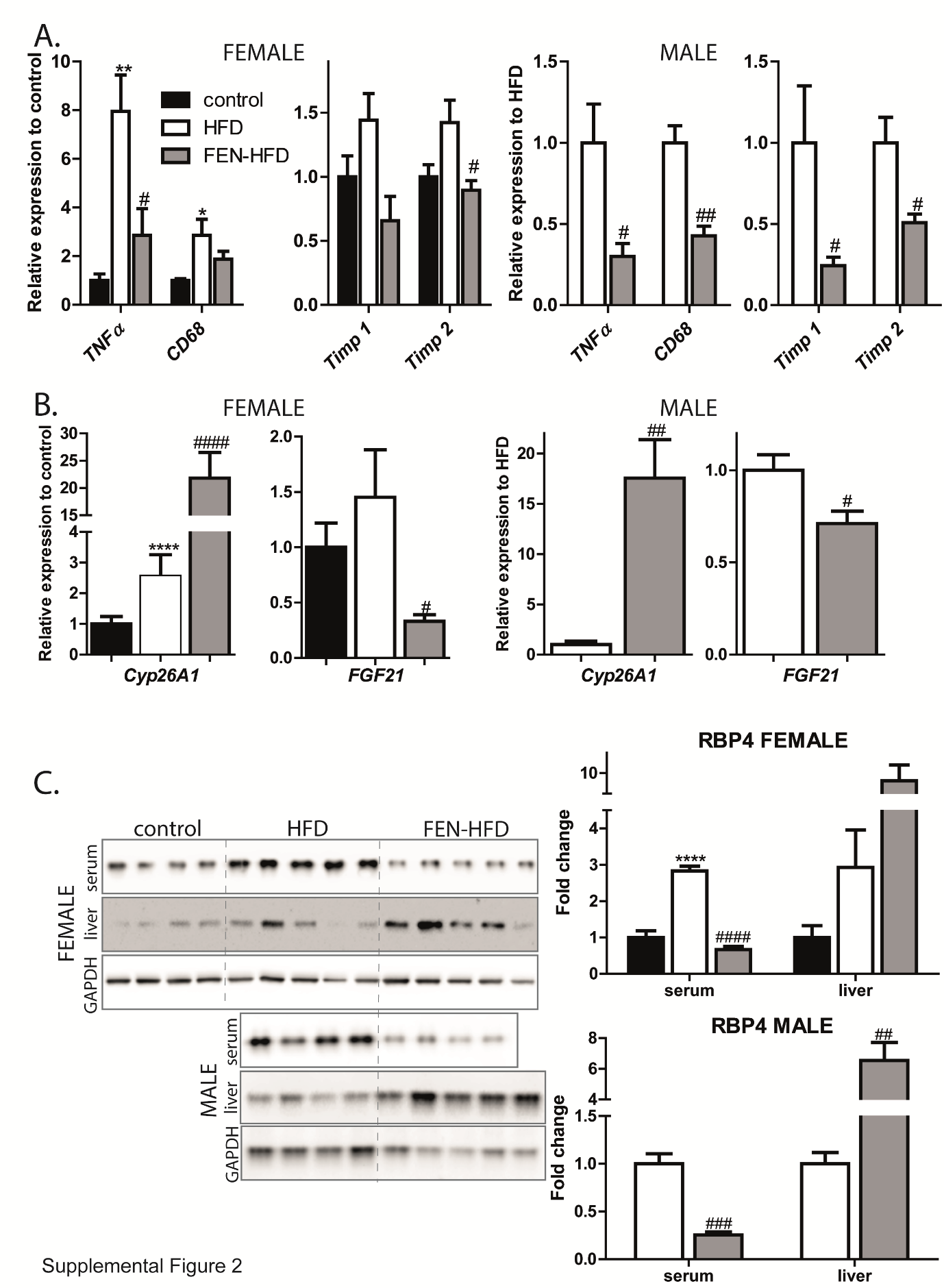
**

**Supplemental Figure 2: Fenretinide alters pro-inflammatory and fibrotic genes and RBP4 levels in male and female ApoE-/- mice.** (**A, B**) Genetic analysis of hepatic tissues in male and female ApoE^-/-^ mice (control n=4, HFD n=5, FEN-HFD n=5). (**C**) Western blot analysis of RPB4 levels in serum and hepatic tissues from female (upper left panels) and male (lower left panels) and quantification (right panels). Data are represented as mean + S.E.M. and analysed by one-way ANOVA followed by Bonferroni multiple comparison t-tests where *p≤0.05, **p≤0.01 and ****p≤0.0001 (compared to control) or #p≤0.05, ##p≤0.01, ### p≤0.001 and #### p≤0.0001 (HFD compared to HFD-FEN).


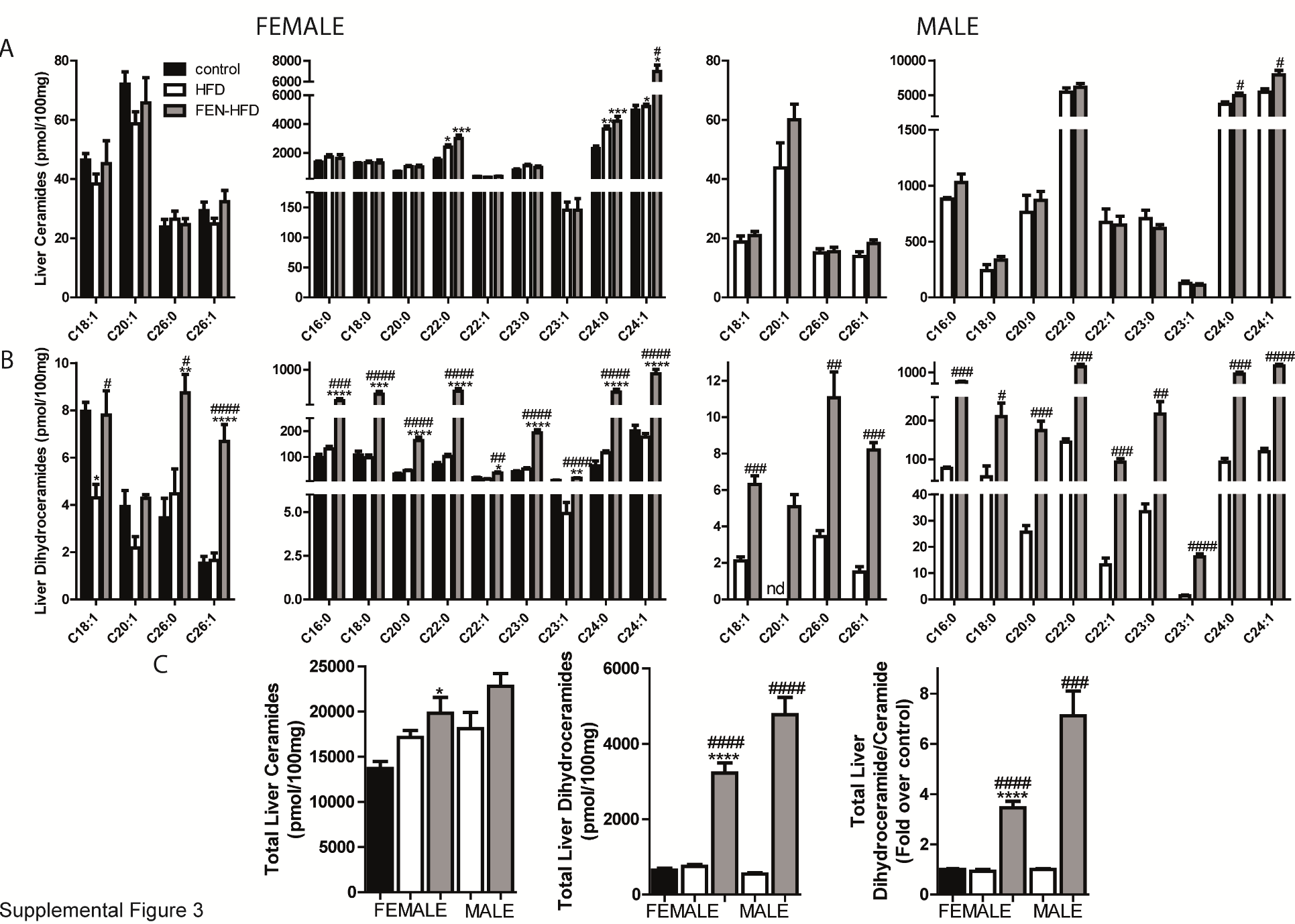


**Supplemental Figure 3: Fenretinide increases dihydroceramide species in hepatic tissues of male and female ApoE^-/-^ mice.** Lipidomic analysis of Ceramide (**A**), Dihydroceramide (**B**) and total (**C**) species in hepatic tissues. Data are represented as mean + S.E.M. and analysed by one-way ANOVA followed by Bonferroni multiple comparison t-tests where *p≤0.05, **p≤0.01, ***p≤0.001 and ****p≤0.0001 (control compared to HFD) or #p≤0.05 and ## p≤0.01, ### p≤0.001 and #### p≤0.001 (HFD compared to FEN-HFD).

**
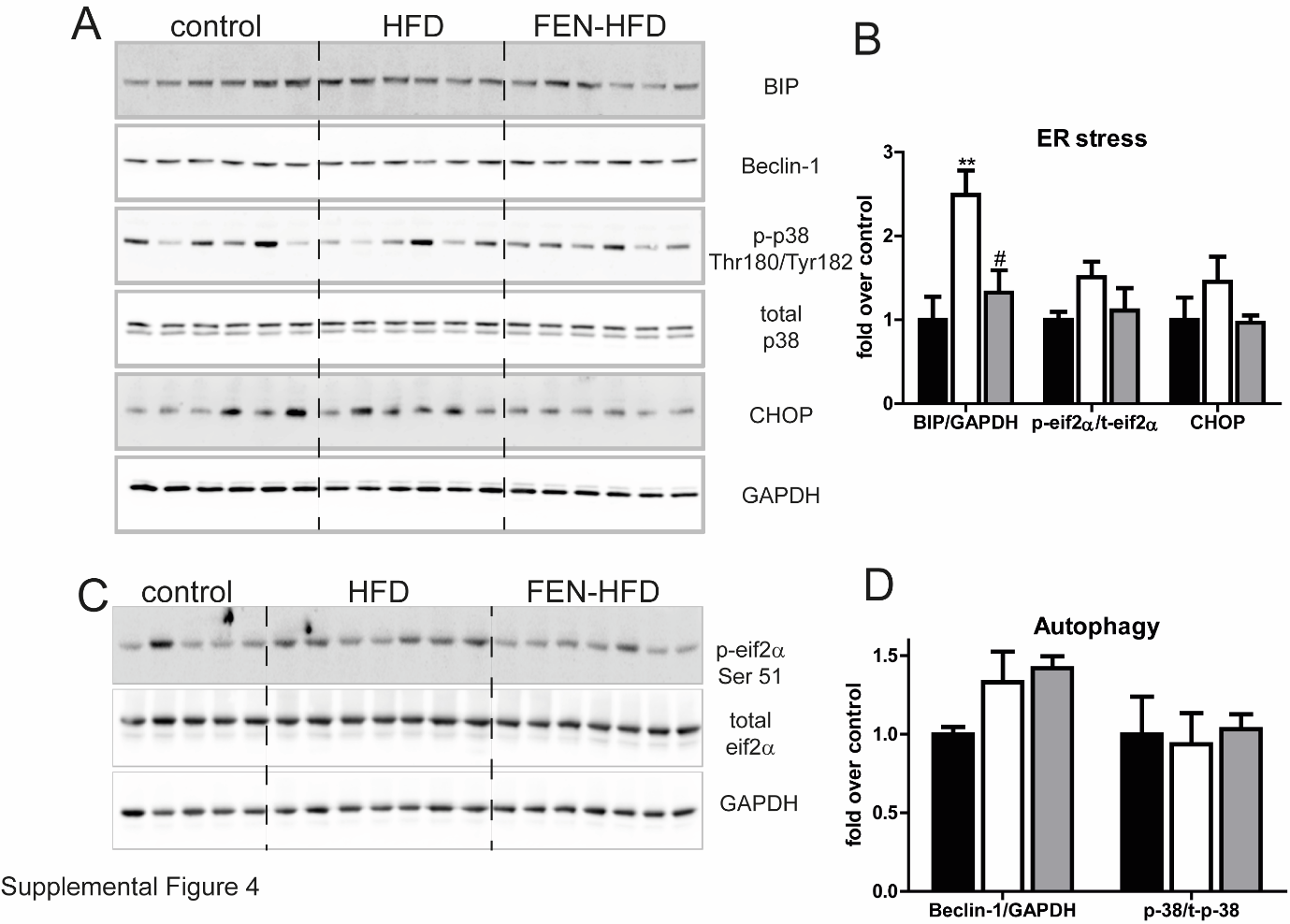
Supplemental Figure 4: Fenretinide alters hepatic ER Stress but not autophagy.** (**A, C**) Western blot analysis of hepatic tissues probed for BIP, Beclin-1 p-eif2α (Ser51), total eif2α, p-p38 (Thr 180/Tyr 182), total p38 or CHOP. GAPDH was used as a loading control. (**B, D**) Quantification of proteins shown in (**A, B**). Data are represented as mean + S.E.M. and analysed by one-way ANOVA followed by Bonferroni multiple comparison t-tests where **p≤0.01 (control compared to HFD) or #p≤0.05 (HFD compared to FEN-HFD).

**
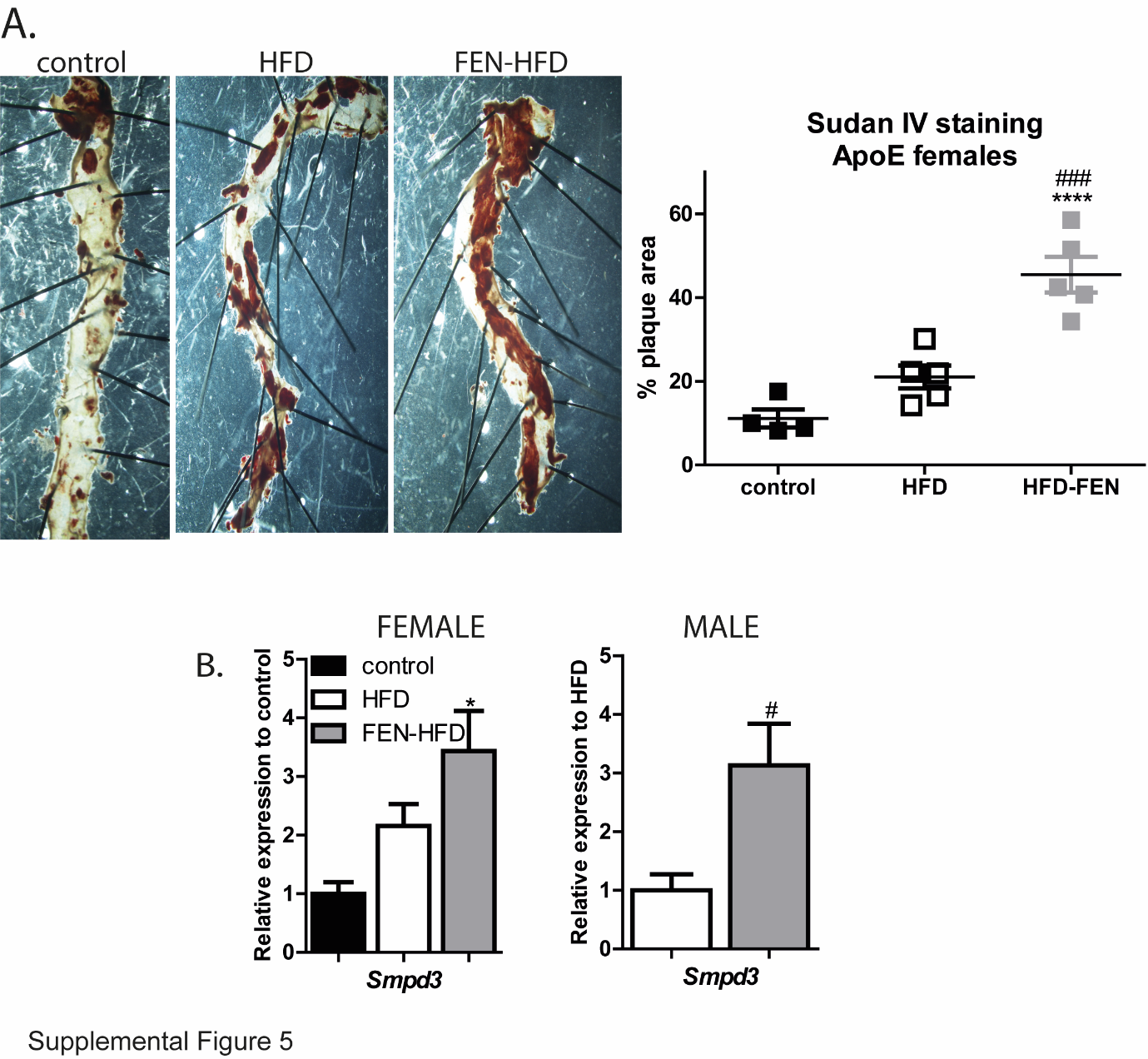
Supplemental Figure 5: Fenretinide increases atherosclerotic plaque formation in female ApoE^-/-^ mice.** The descending aorta was prepared *en face* from (**A**) female ApoE^-/-^ mice and stained with Sudan IV and quantified using Image J. Representative images are shown. Genetic analysis of *Smpd3* levels in hepatic tissue of female (**B**) and male (**C**) ApoE^-/-^ mice. Data are represented as mean $\pm$ S.E.M. (n=3-5 per group) and analysed by one-way ANOVA followed by Bonferroni multiple comparison t-tests where *p≤0.05 and ****p≤0.0001 (compared to control) or # p≤0.05 and ### p≤0.001 (HFD compared to HFD-FEN).

A

B

**Supplemental Figure 6: Fenretinide does not induce splenomegaly in LDLR^-/-^ but does in ApoE^-/-^ mice.** Individual spleens were weighed and normalised for body weight of individual mice. Data are represented as mean $\pm$ S.E.M. (A. n=14-18 per group LDLR^-/-^ mice; B. n=4-5 per group ApoE^-/-^ mice) and analysed by one-way ANOVA followed by Bonferroni multiple comparison t-tests where **p≤0.01 (compared to control), # p≤0.05 and ## p≤0.01 (HFD compared to HFD-FEN) and $$ p≤0.01 (ApoE^-/-^ FEN-HFD, male compared to female).


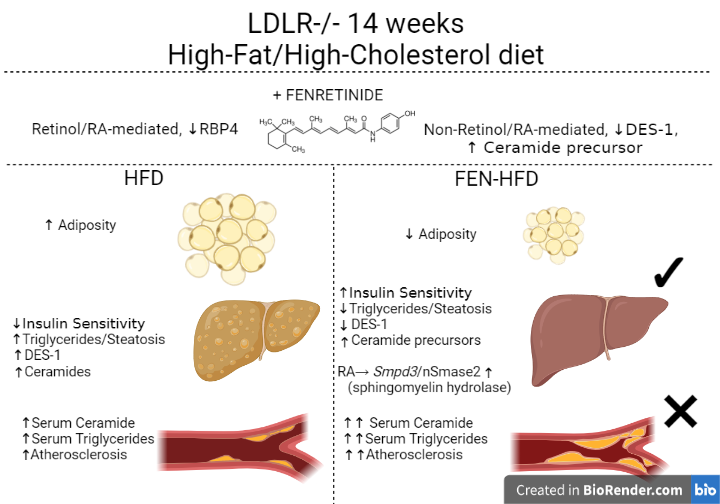


**Supplemental Figure 7: Working Model of Fenretinide Action.**

A graphical representation demonstrating the effects of FEN on multiple metabolic complications using LDLR^-/-^ mice fed high-fat/high-cholesterol diet +/- FEN as a model of atherosclerosis and non-alcoholic fatty liver disease (NAFLD). FEN treatment prevented excess weight gain and adiposity, improved insulin sensitivity and completely inhibited hepatic triglyceride accumulation, ballooning and steatosis. In association, FEN decreased the expression of hepatic genes driving NAFLD, inflammation and fibrosis e.g., *Hsd17b13*, *Cd68* and *Col1a1*. The mechanisms of FEN’s beneficial effects in association with decreased adiposity were mediated by suppression of ceramide synthesis, via hepatic DES1 protein, leading to increased ceramide precursors (i.e. dihydroceramides). However, FEN treatment in LDLR^-/-^ mice enhanced circulating triglycerides and accelerated aortic plaque formation. FEN increased hepatic sphingomyelinase *Smpd3* expression, via a retinoic acid mediated mechanism, linking induction of an alternative ceramide generation pathway (via sphingomyelin hydrolysis) to a novel mechanism of increased atherosclerosis. Pharmacological treatment of both DES1 and *Smpd3*/nSMase2 may be a novel, more potent therapeutic approach for the treatment of metabolic syndrome.

**Full images of blot membranes**

Gels/blots used in figures are all compliant with the digital image and integrity policies of Nature publishing and Scientific Reports journal. See main manuscript methods section for details. Briefly:

Western blot membranes were cut at approximate molecular weight ( +/- 20 kDa ) of target protein before incubation of primary antibodies. The following are full images of blot membranes from a high-sensitivity CCD camera system, overexposed to visualise edges of membranes. Due to the high signal-to-noise ratio with some antibodies and the nature of the high-sensitivity camera system, the edge of the membrane is often not visible, even when altering the exposure, contrast and brightness.


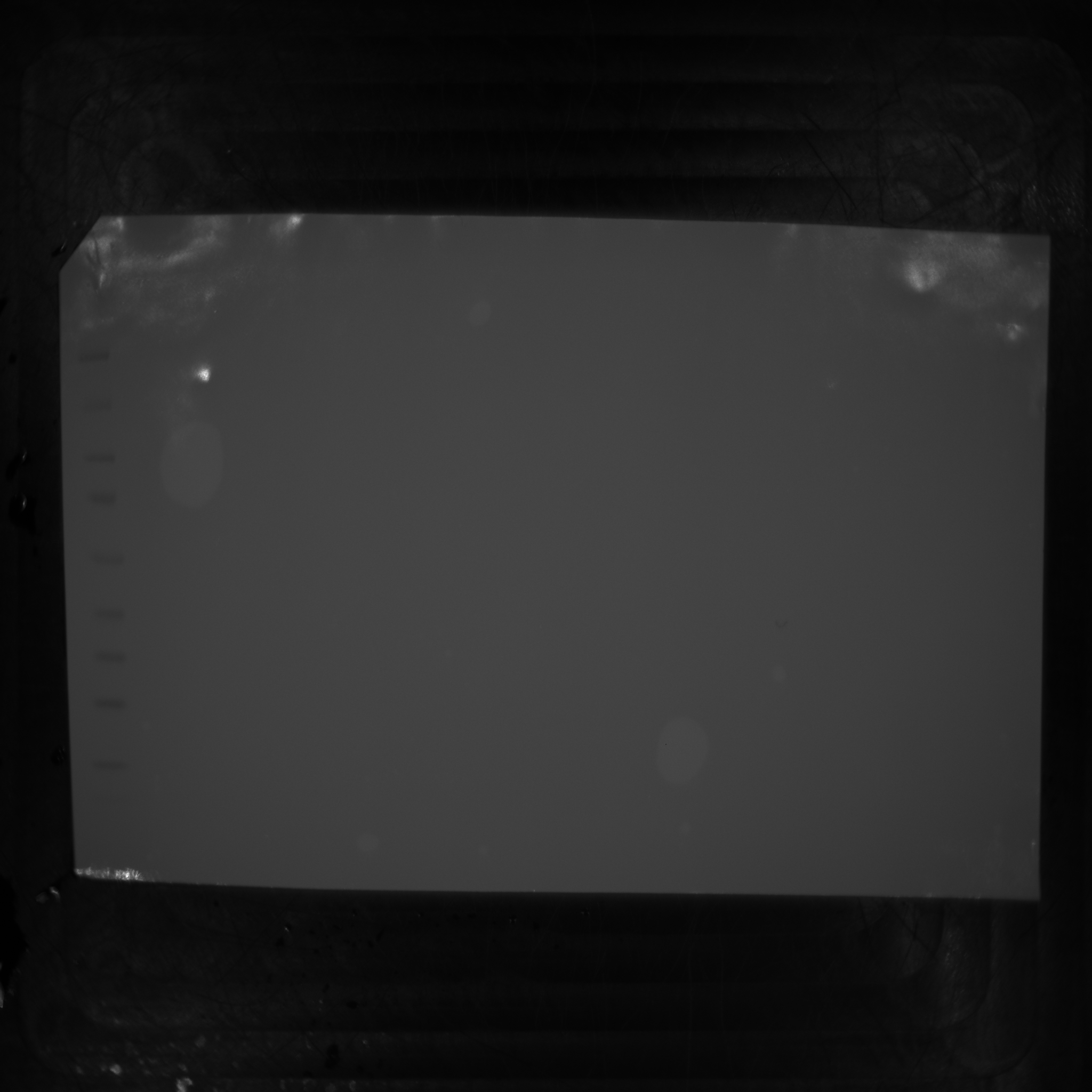

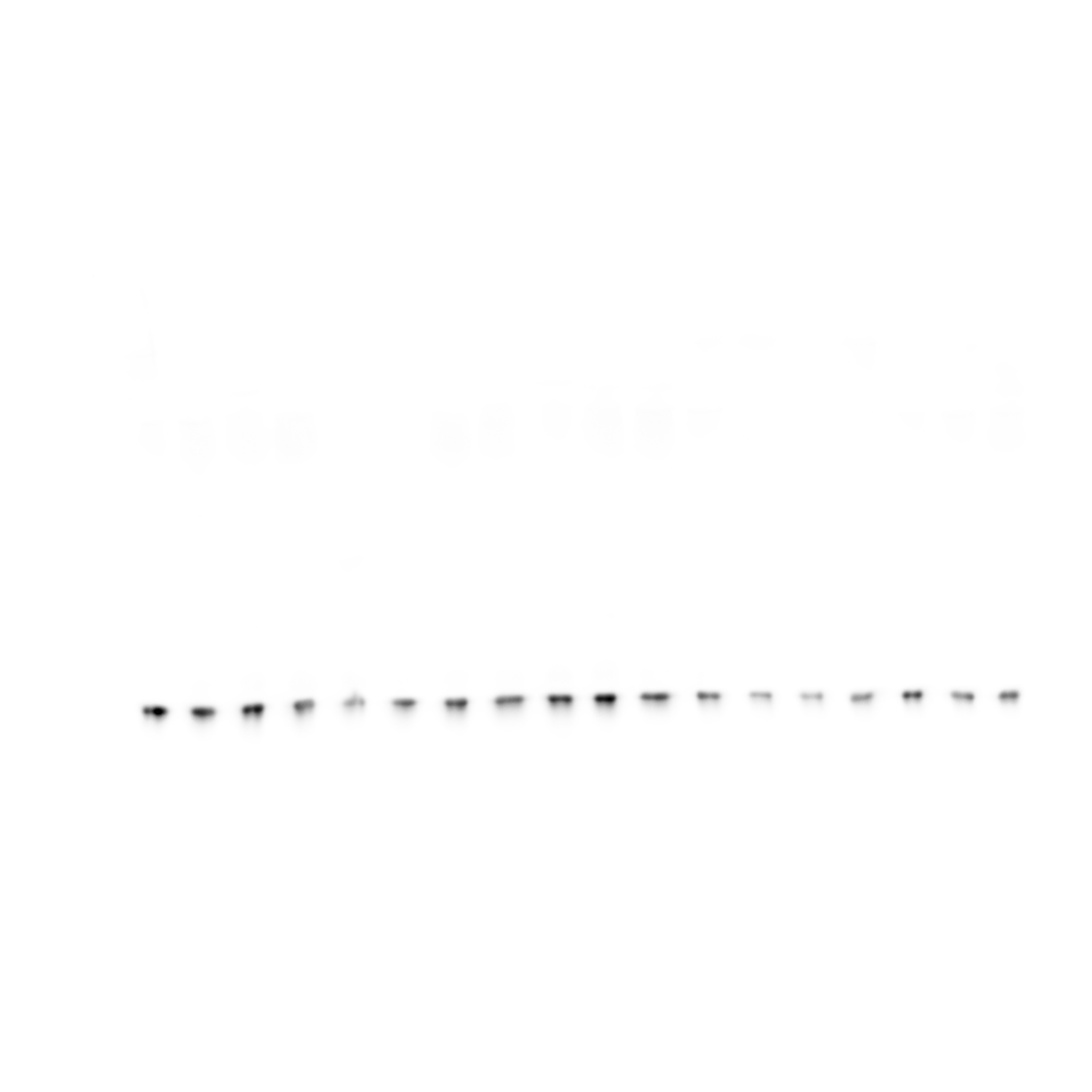
Figure 1D serum RBP4 (21 kDa, uncut membrane, 4-12% Bis-Tris gel transferred to membrane)

RBP4 ladder image

RBP4 image

RBP4

50

40

35

25

15

70

100

140

260

Figure 1F Liver insulin signalling (4-12% Bis-Tris gel transferred to membrane),
plus Muscle insulin signalling (Supp Fig 1B)


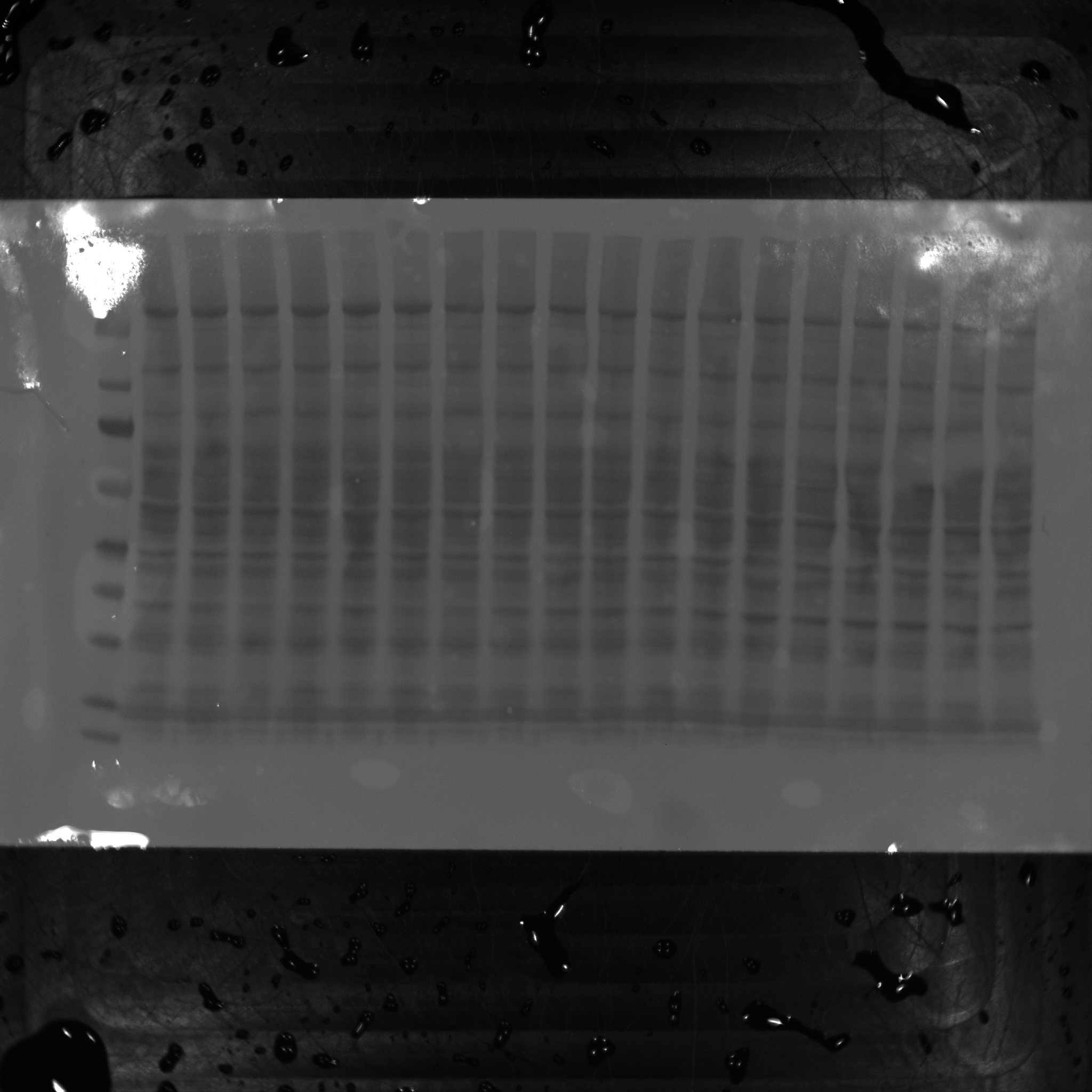


50

40

35

25

15

70

100

140

260

Total IR

Akt

GSK3β

S6

GAPDH


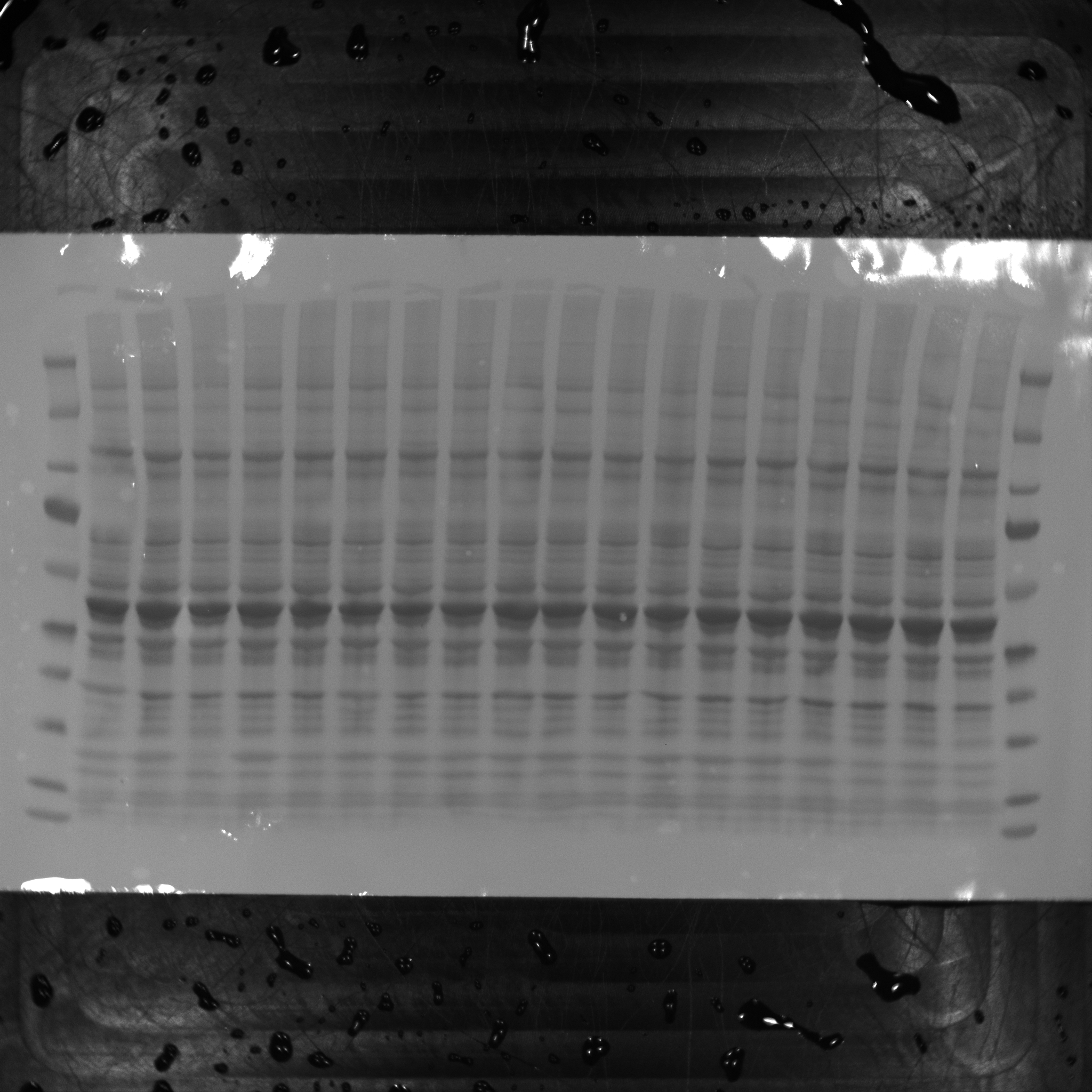


Dashed lines indicate where membrane was cut after ponceau S Staining

Liver membrane ponceau - Figure 1F

Muscle membrane Ponceau
Supp Figure 1B

50

40

35

25

15

70

100

140

260

Total IR

Akt

GSK3β

S6

GAPDH

Figure 1F liver IR β-chain (total protein, 95 kDa, top of cut membrane)


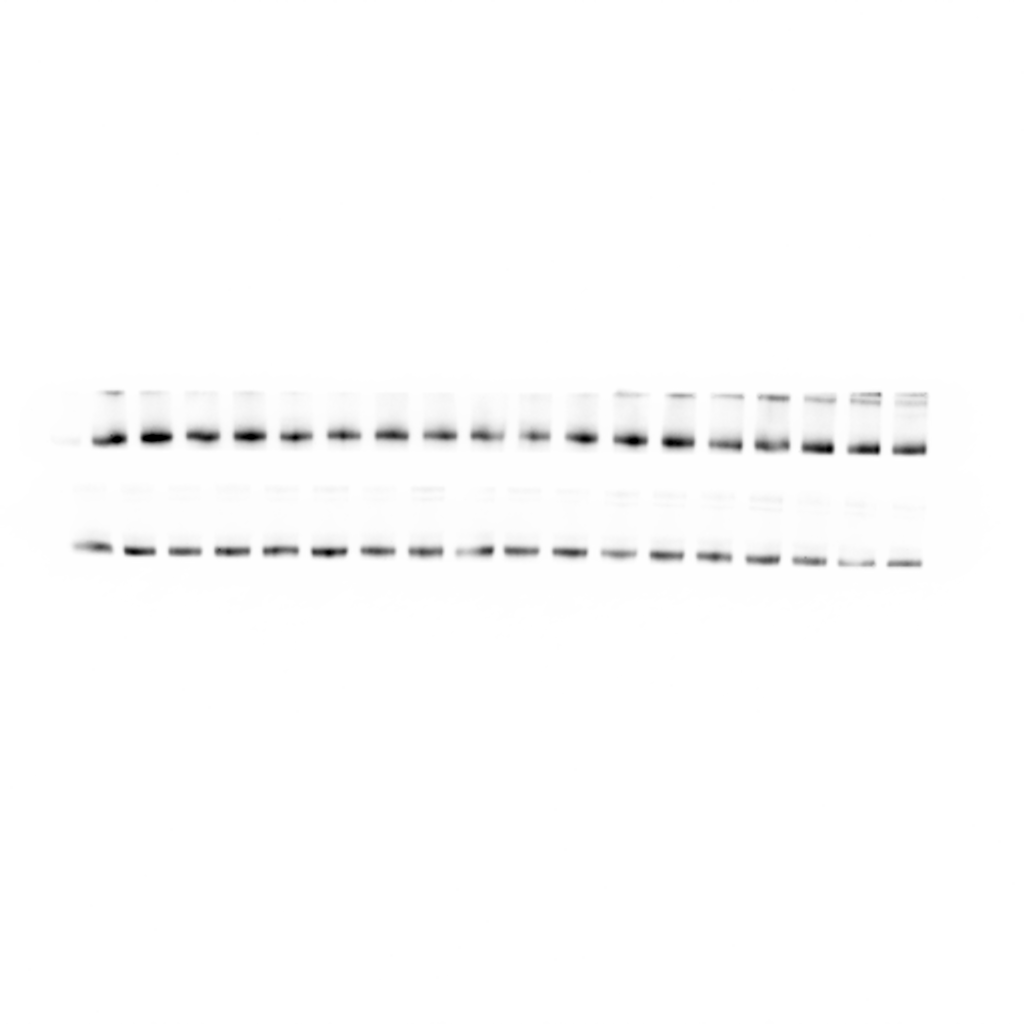
Figure 1F liver Akt (phospho Ser 473, 60 kDa, middle of cut membrane)

100

Total IR

100

liver

muscle

Upper panel Figure 1F

Lower panel Supp Figure 1B


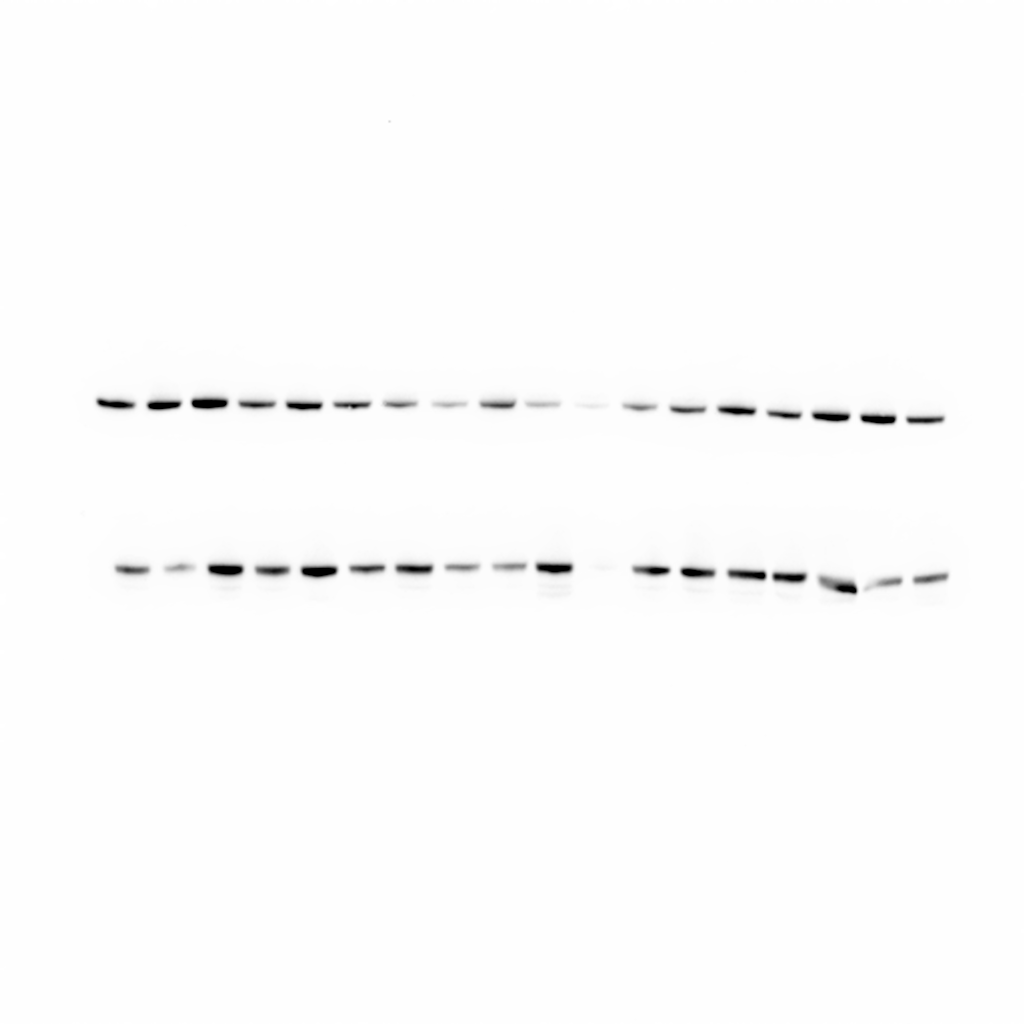


50

70

50

70

liver

muscle

pAkt

Upper panel Figure 1F

Lower panel Supp Figure 1B

Figure 1F liver Akt (total protein, 60 kDa, middle of cut membrane)


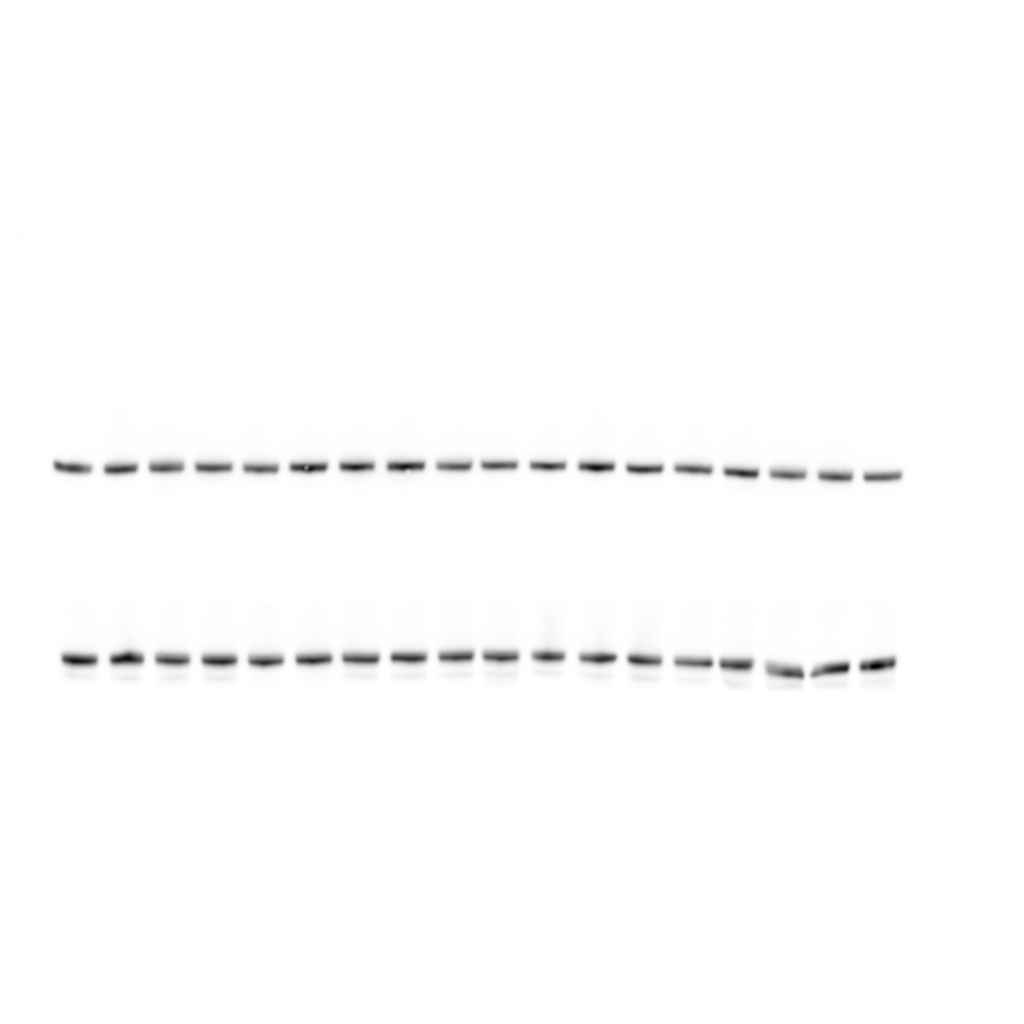


50

70

50

70

Total Akt

liver

muscle

Upper panel Figure 1F

Lower panel Supp Figure 1B

Figure 1F liver GSK3β (phospho Ser 9, 46 kDa, bottom of cut membrane)


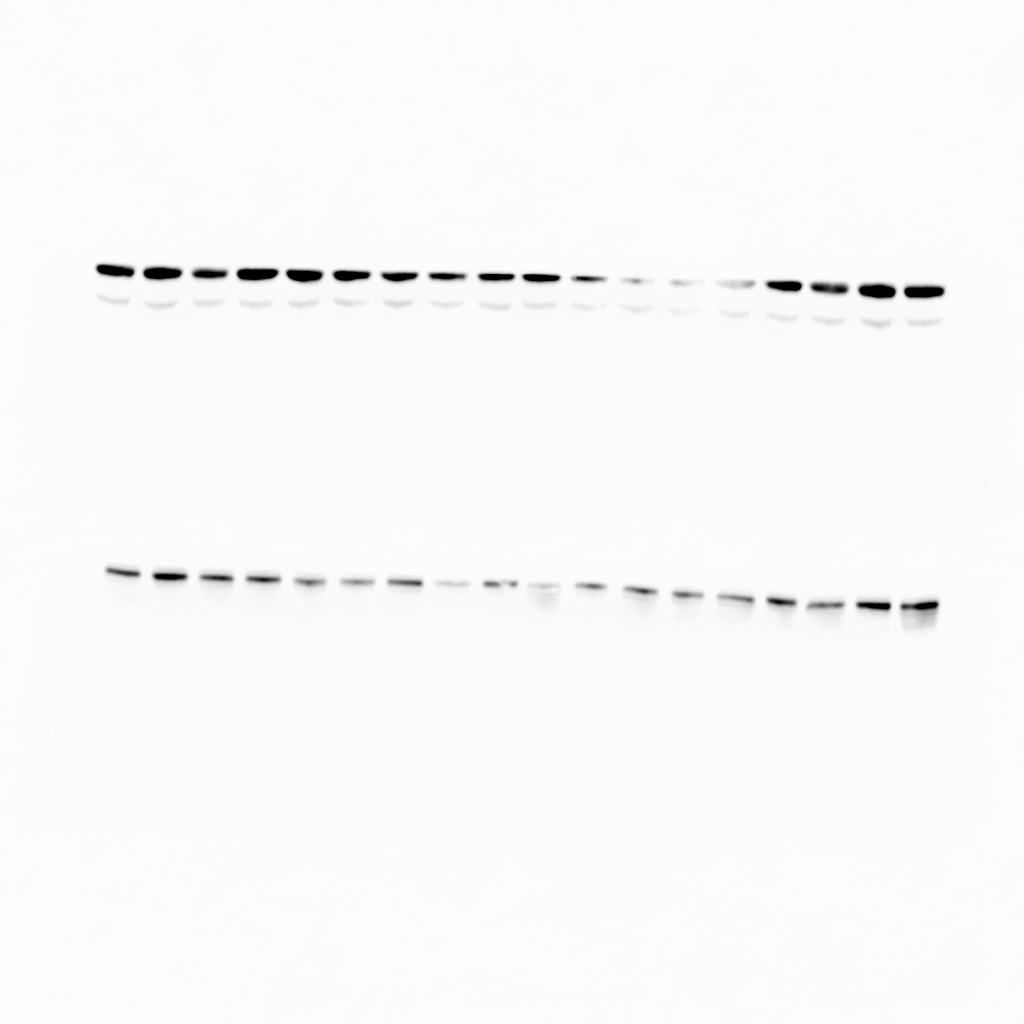


40

35

25

15

35

25

15

40

liver

muscle

pGSK3β

Upper panel Figure 1F

Lower panel Supp Figure 1B

Figure 1F liver S6 (phospho Ser 235/236, lower band, 32 kDa, bottom of cut membrane)

Figure 1F liver S6 (total protein, lower band, 32 kDa, bottom of cut membrane)


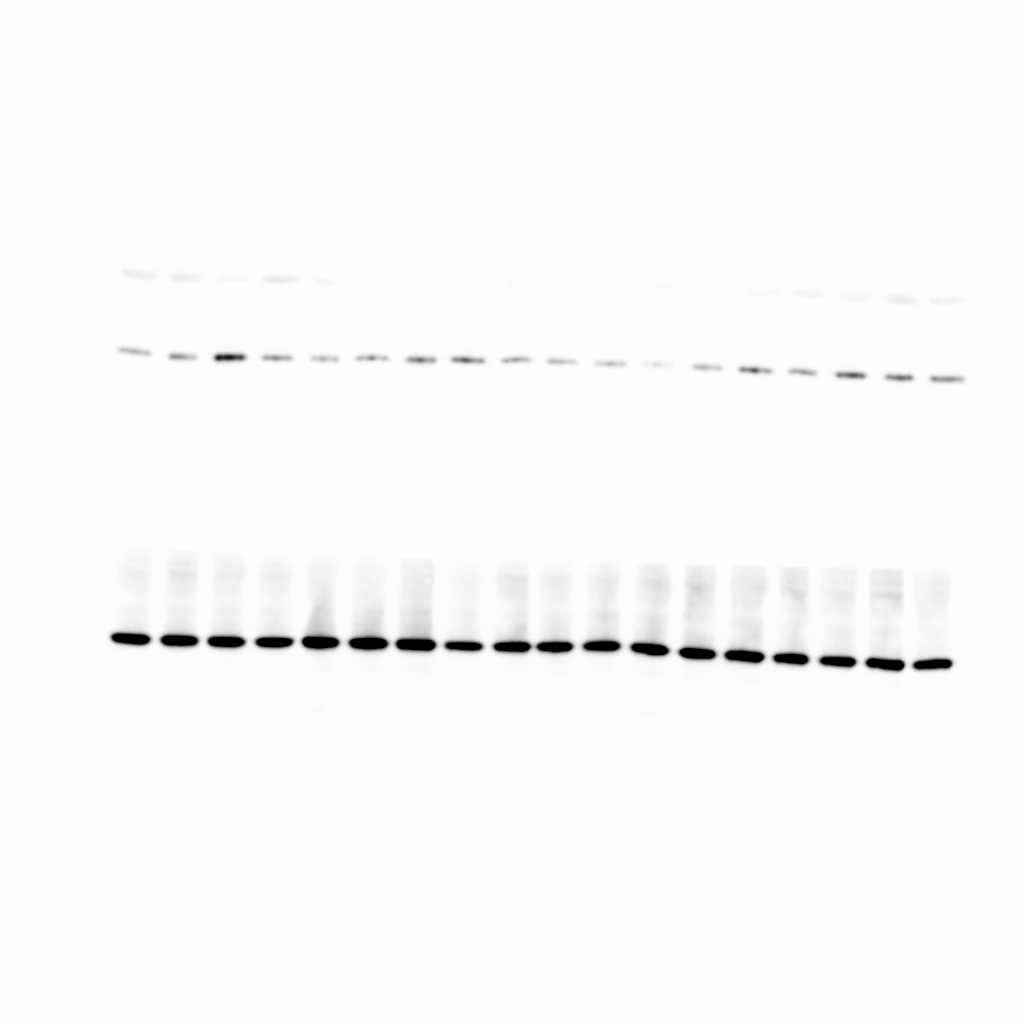

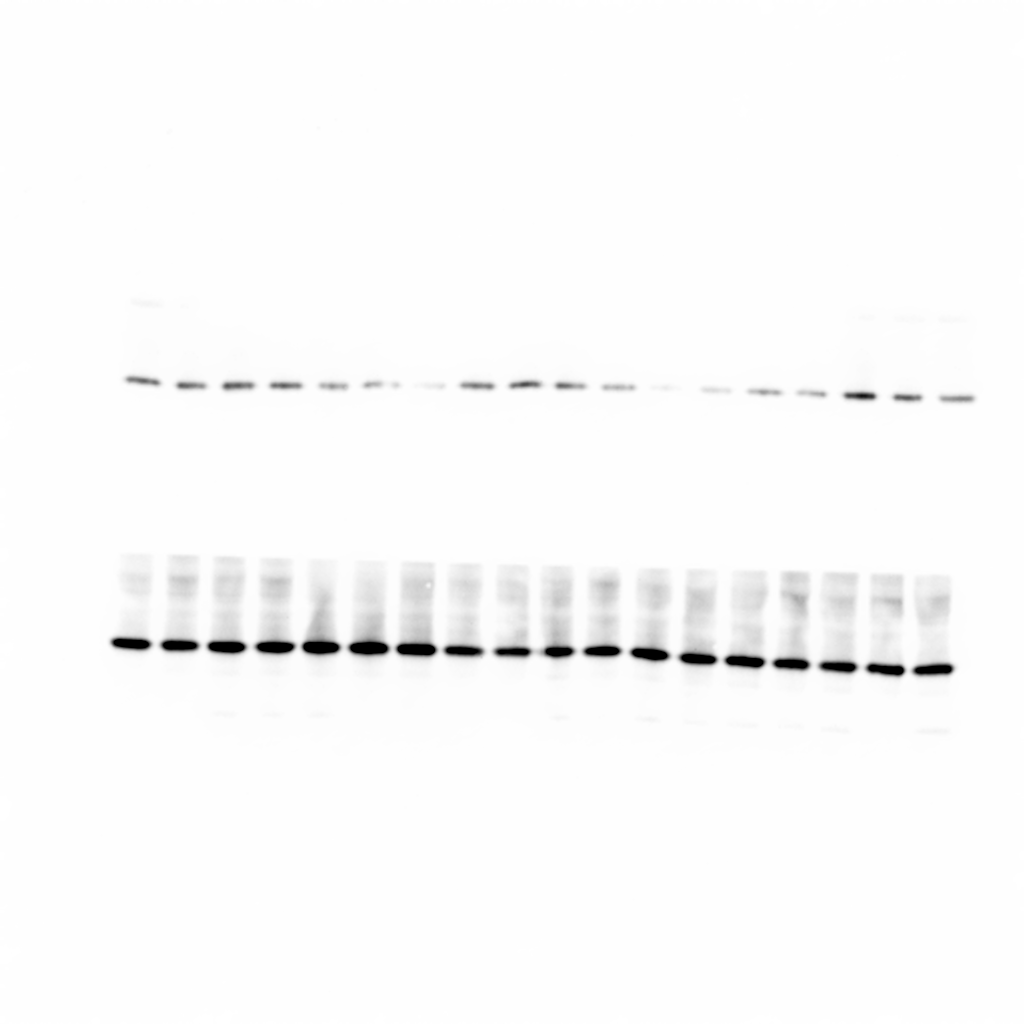


40

35

25

15

35

25

15

40

liver

muscle

pS6

40

35

25

15

35

25

15

40

Total S6

Upper panel Figure 1F

Lower panel Supp Figure 1B

Upper panel Figure 1F

Lower panel Supp Figure 1B

Figure 1F liver GAPDH (total protein, upper band, 37 kDa, stripped and reprobed from total S6 above, bottom of cut membrane)


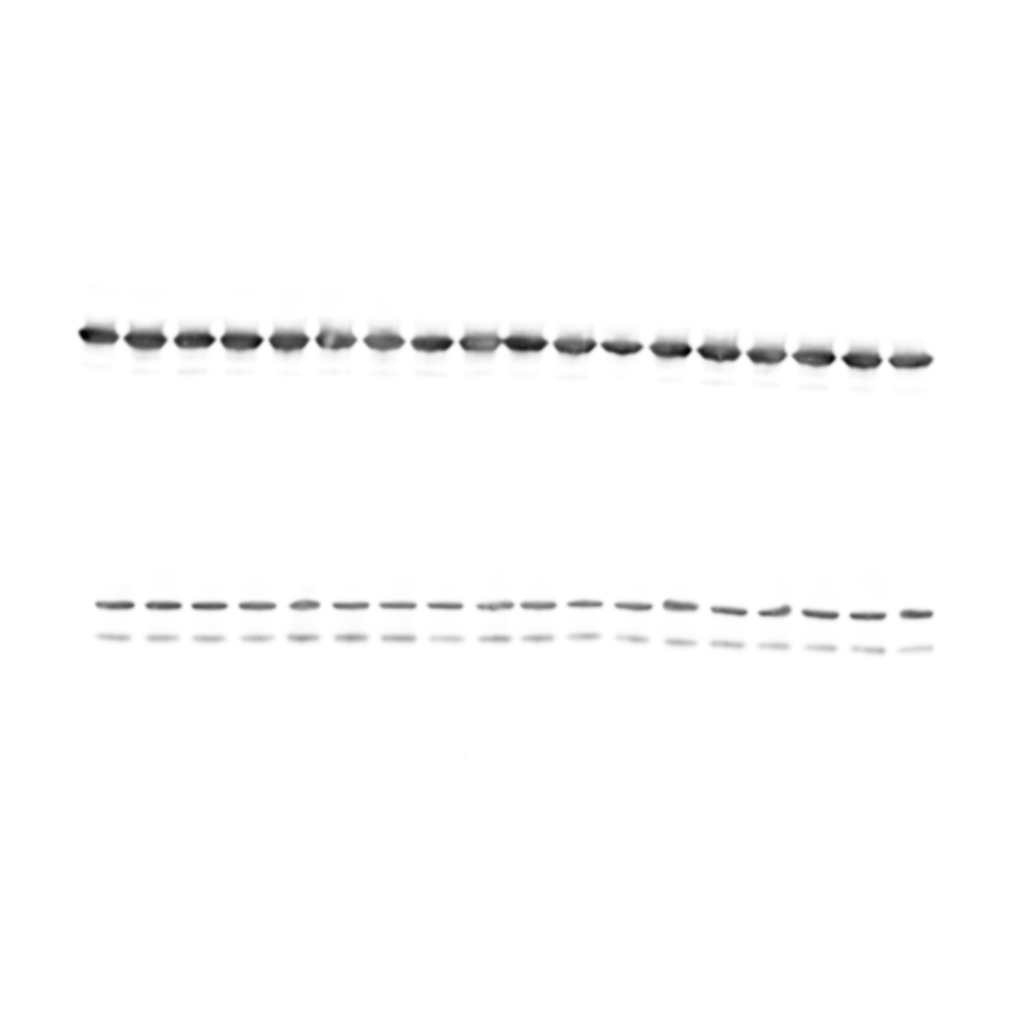


40

35

25

15

35

25

15

40

liver

muscle

GAPDH

Upper panel Supp Figure 1B

Lower panel Figure 1F

Figure 4A liver DEGS1 (total protein, ~34 kDa, bottom of cut membrane, 4-12% Bis-Tris gel transferred to membrane)
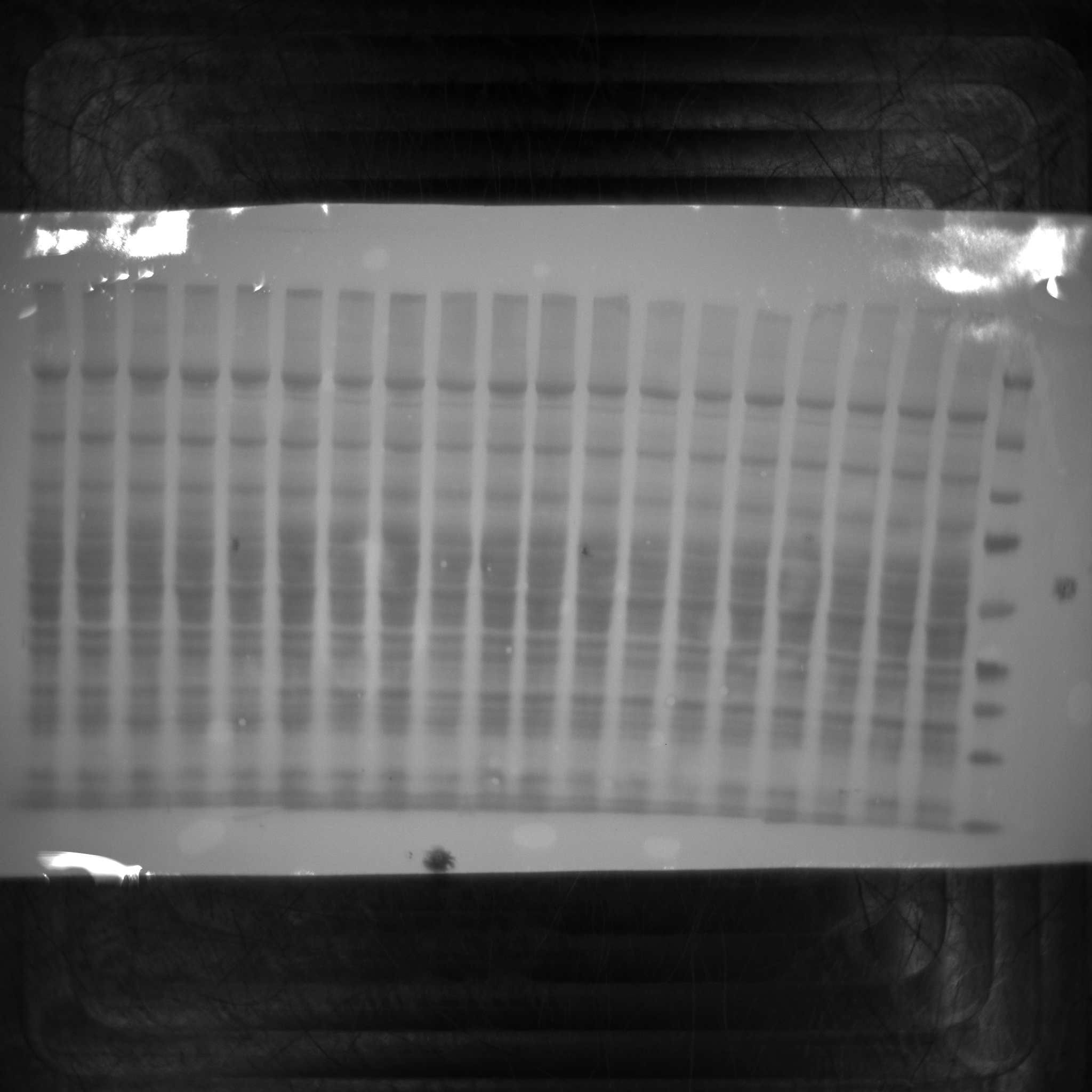

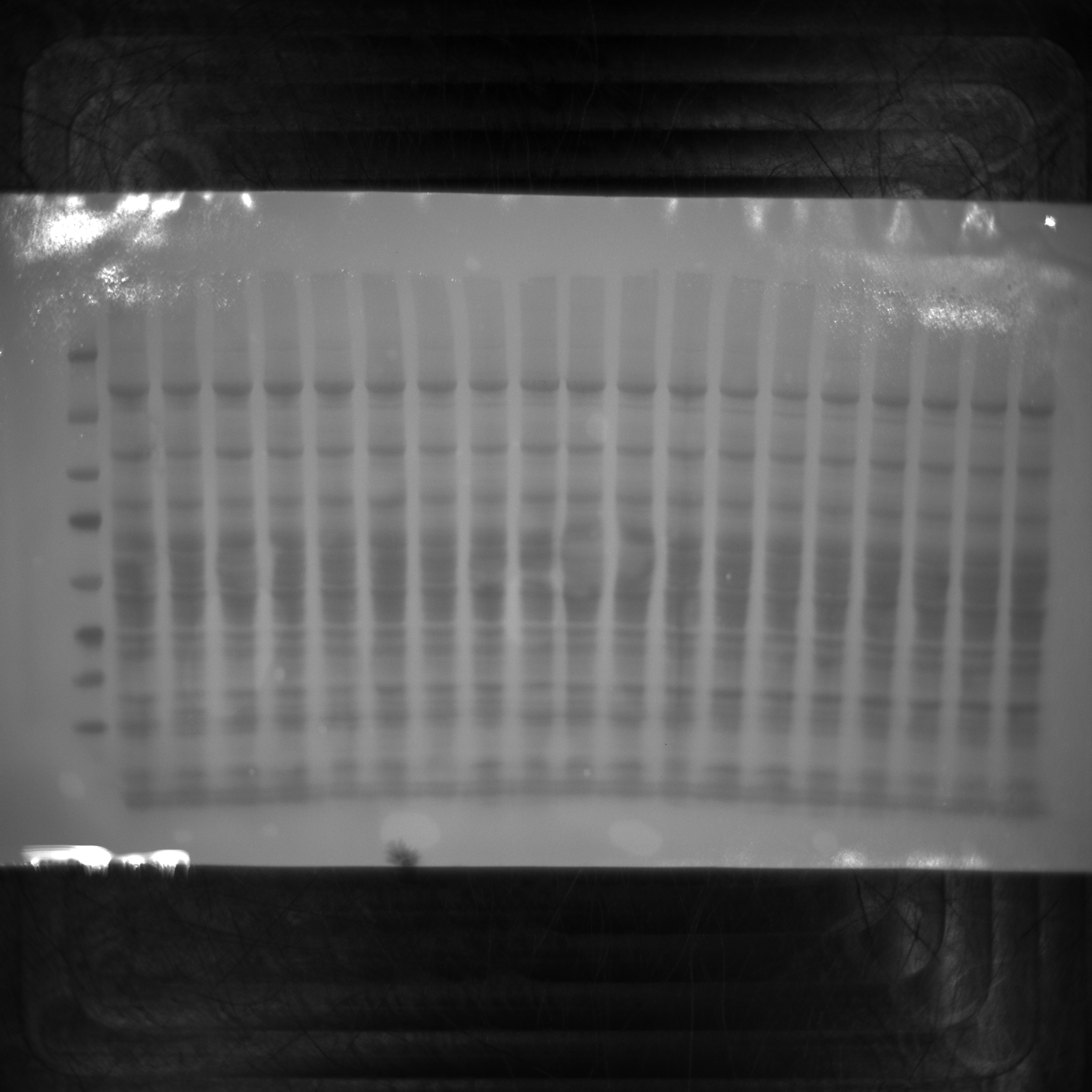


Liver membrane Ponceau gel 2

Liver membrane Ponceau gel 1

Dashed lines indicate where membrane was cut after ponceau S Staining

Figure 4A

Gel 2 used as ‘representative’ image in figure

Quantification from both

50

40

35

25

15

70

100

140

260

50

40

35

25

15

70

100

140

260

DES1/GAPDH

Figure 4A


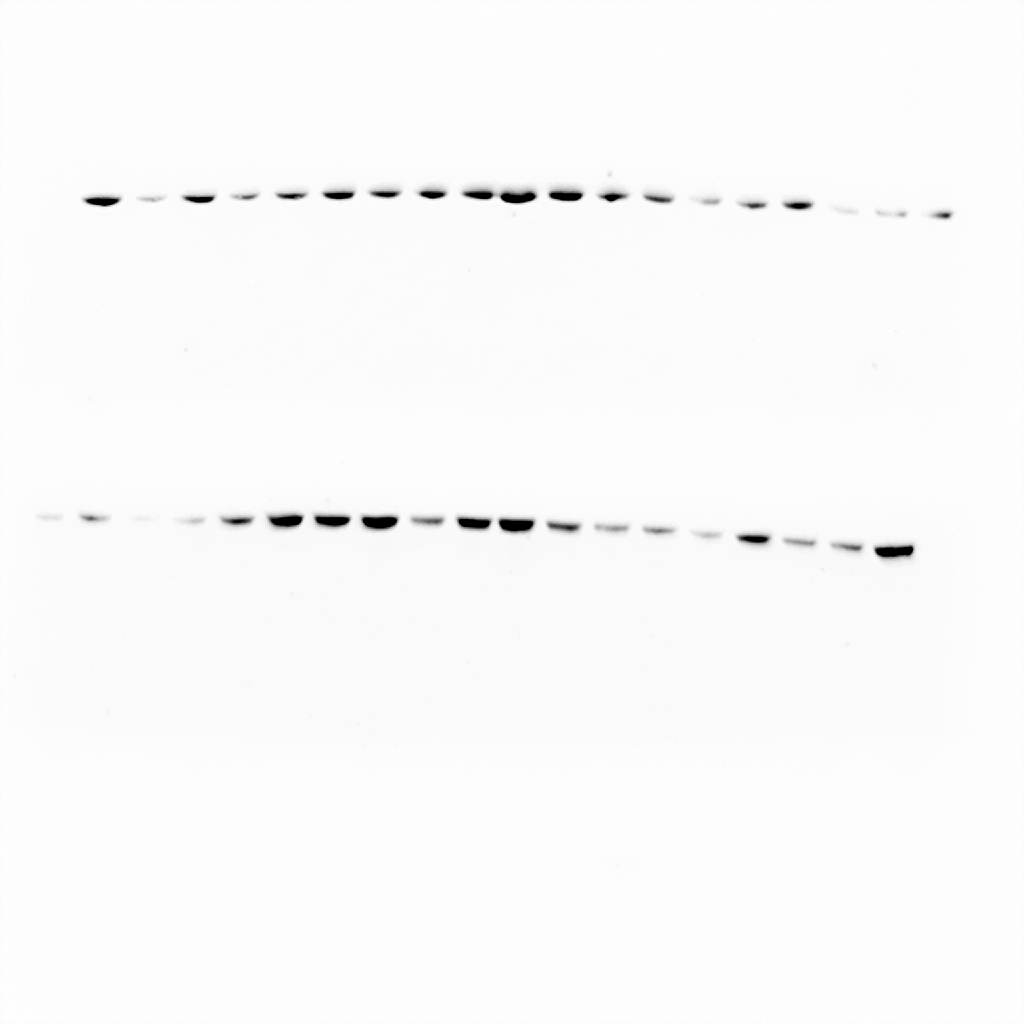


50

40

35

25

15

50

40

35

25

15

DES1

Figure 4A

Lower blot ‘representative’ image in figure

Figure 4A liver GAPDH (total protein, 37 kDa top band, stripped and reprobe of DES1 membrane above, bottom of cut membrane, 4-12% Bis-Tris gel transferred to membrane)


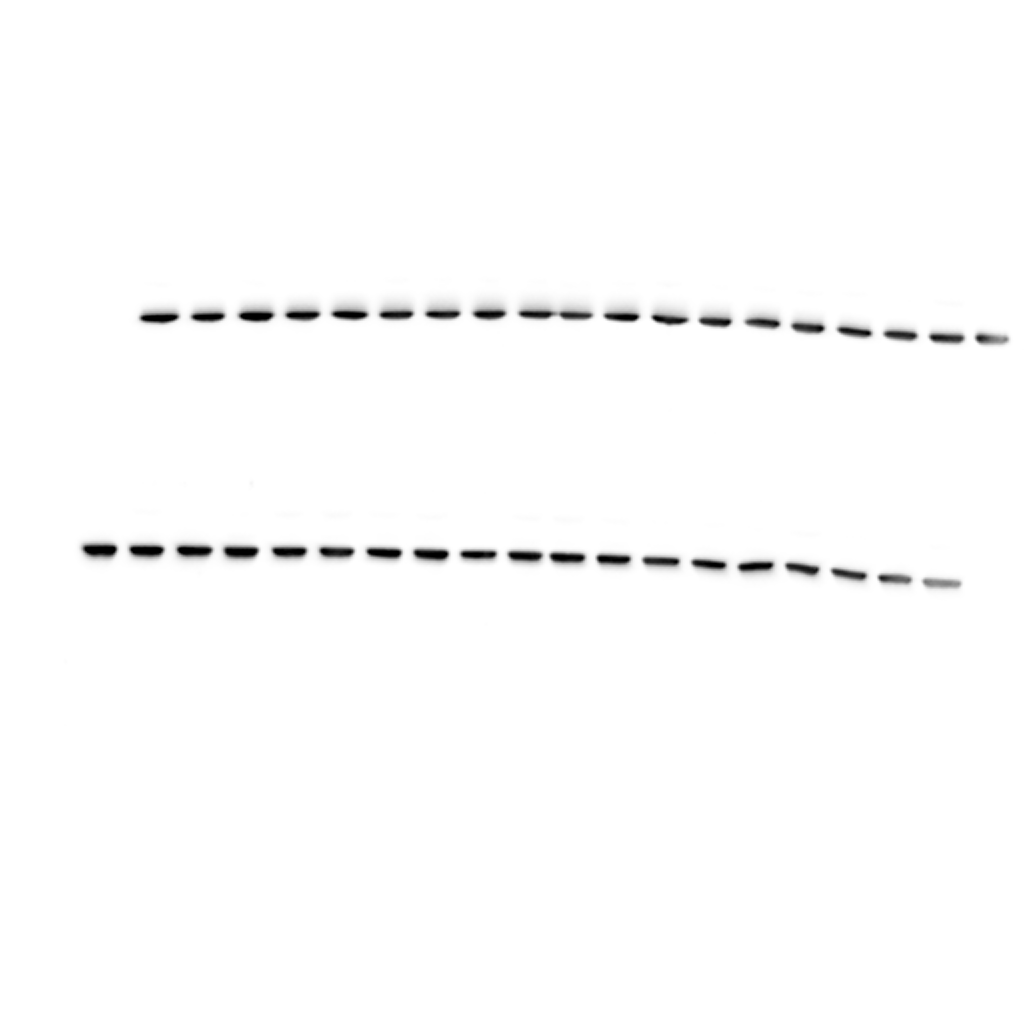


50

40

35

25

15

50

40

35

25

15

GAPDH

Figure 4A

Lower blot ‘representative’ image in figure

Figure 5C


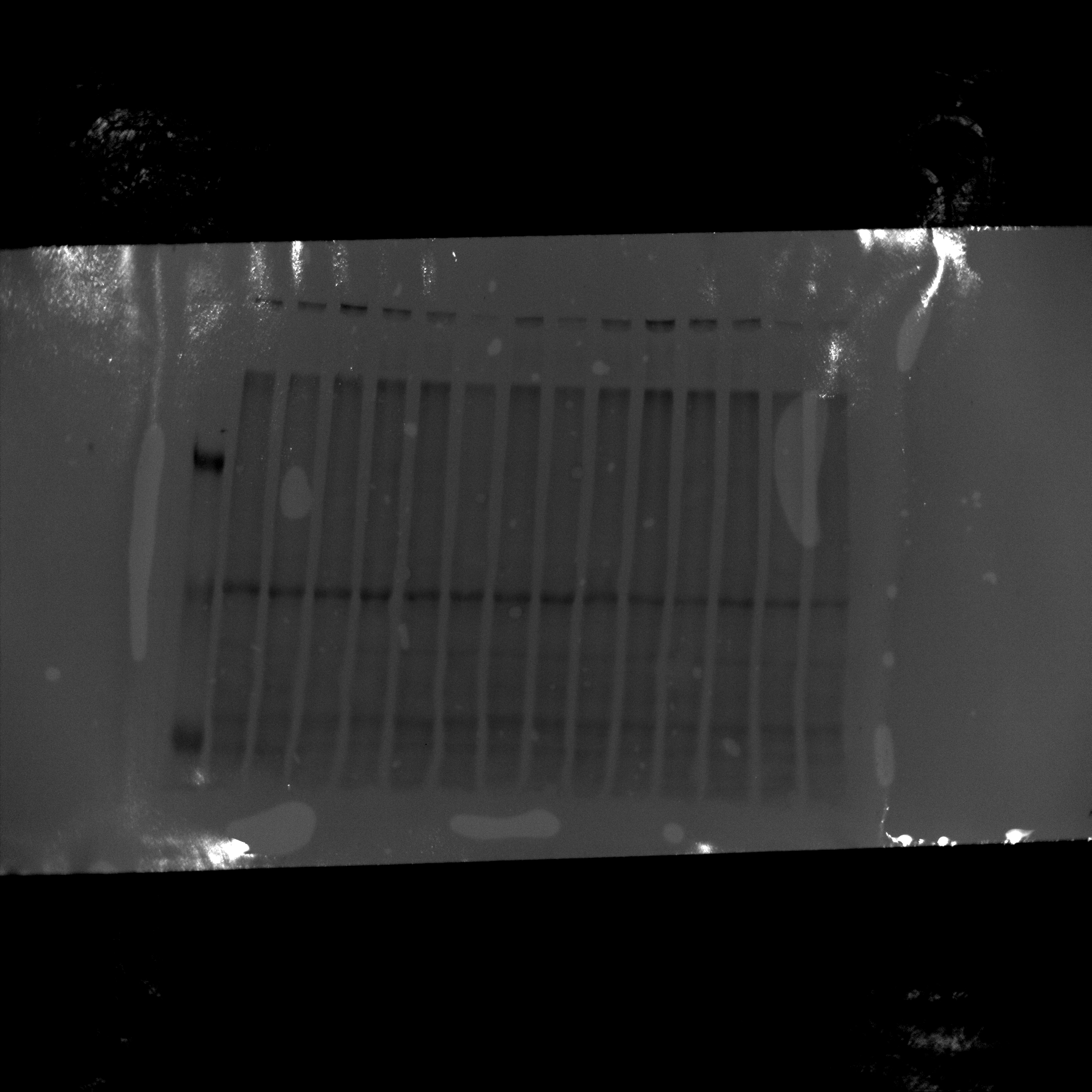

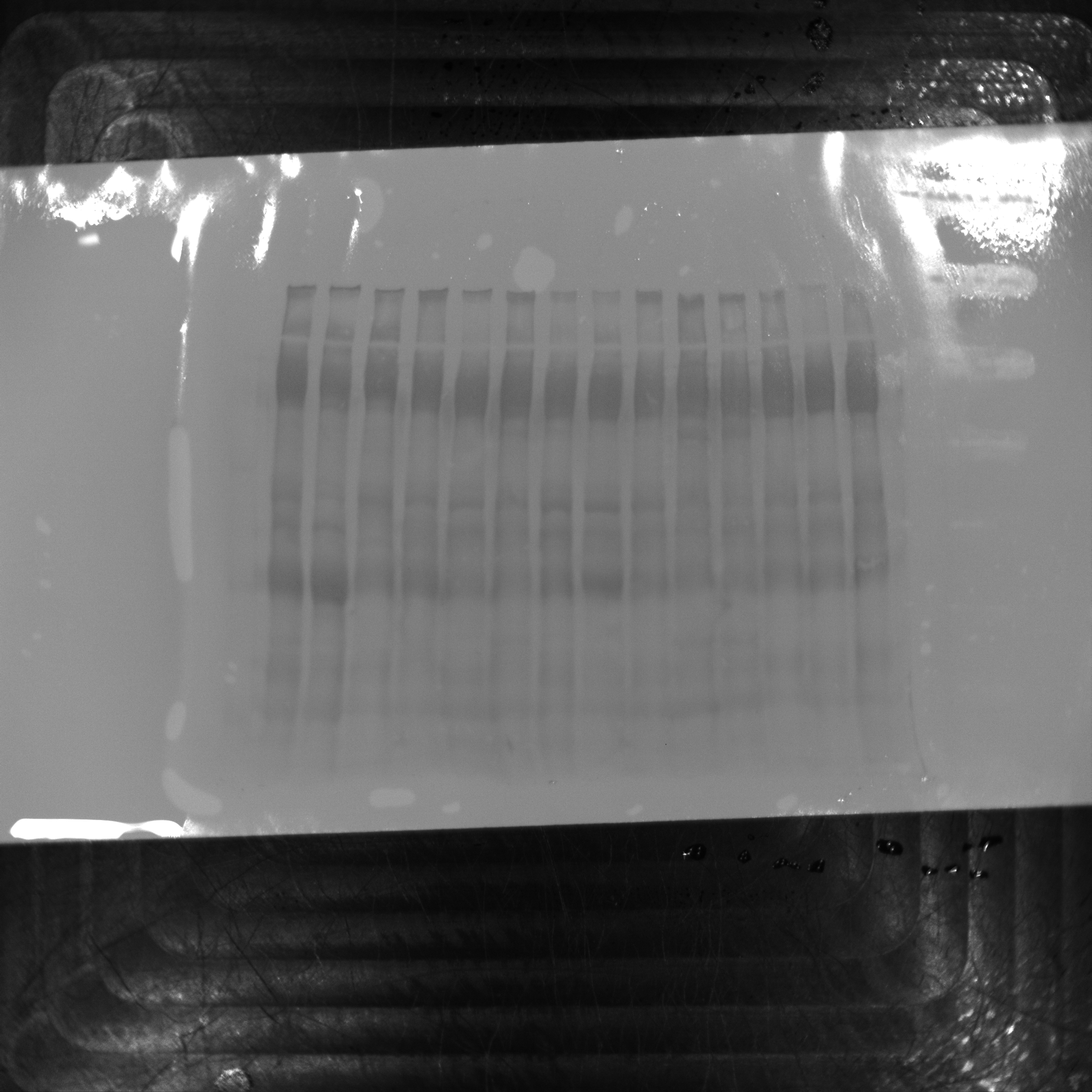


100

140

260

100

140

260

Liver Ponceau

Figure 5C

Serum Ponceau

Figure 5C

Figure 5C

Serum ApoB proteins (total)


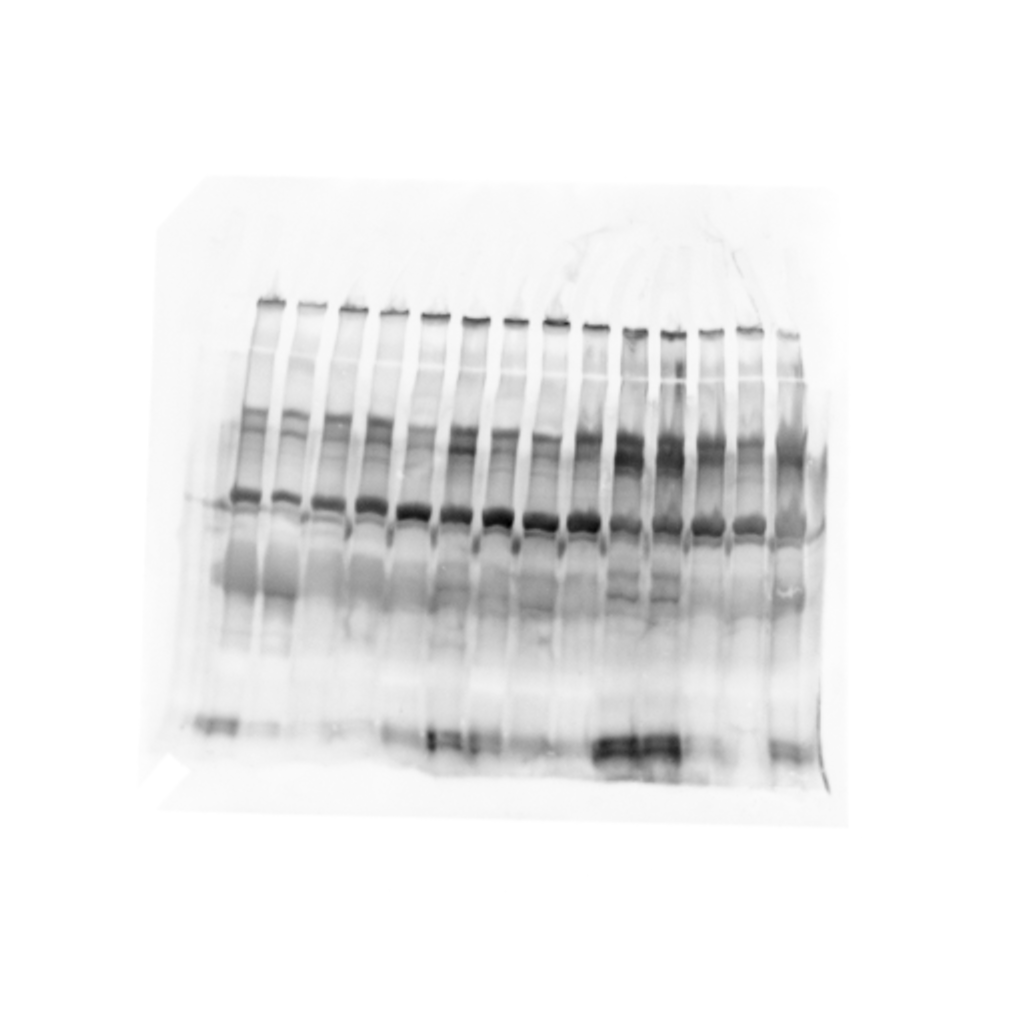


100

140

260

Serum ApoE 100/48

Liver ApoB proteins (total)


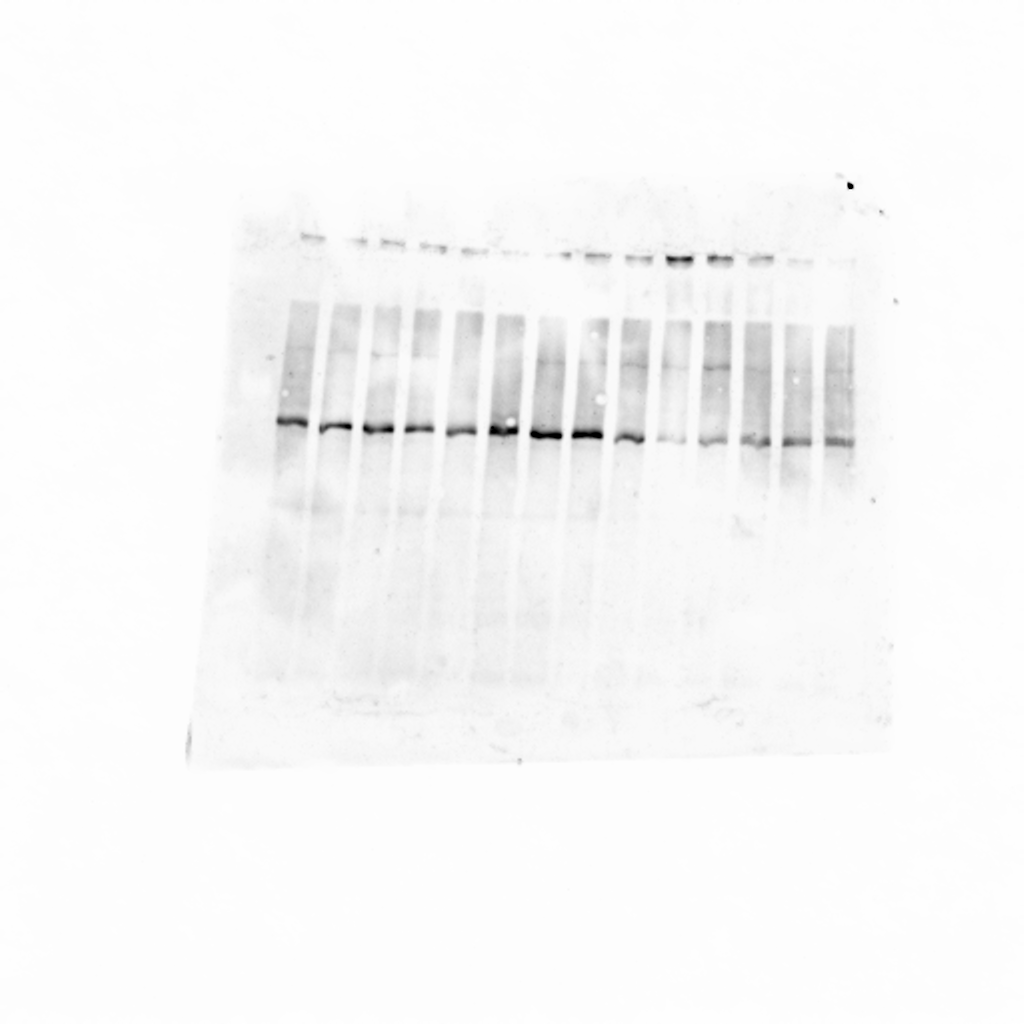


100

140

260

Liver ApoE 100/48

Figure 5C

Liver

Strip and reprobe for Vinculin (bottom band)


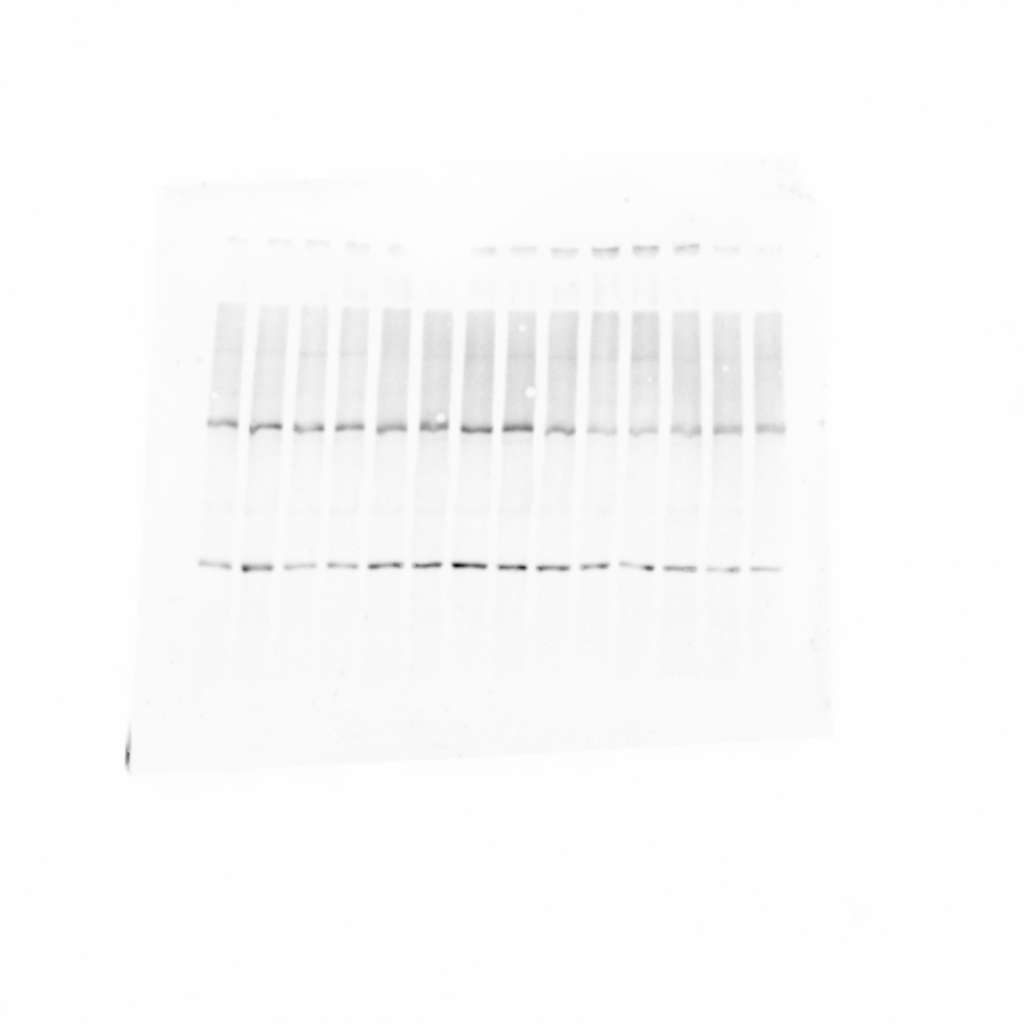


100

140

260

Vinculin ApoE 100/48


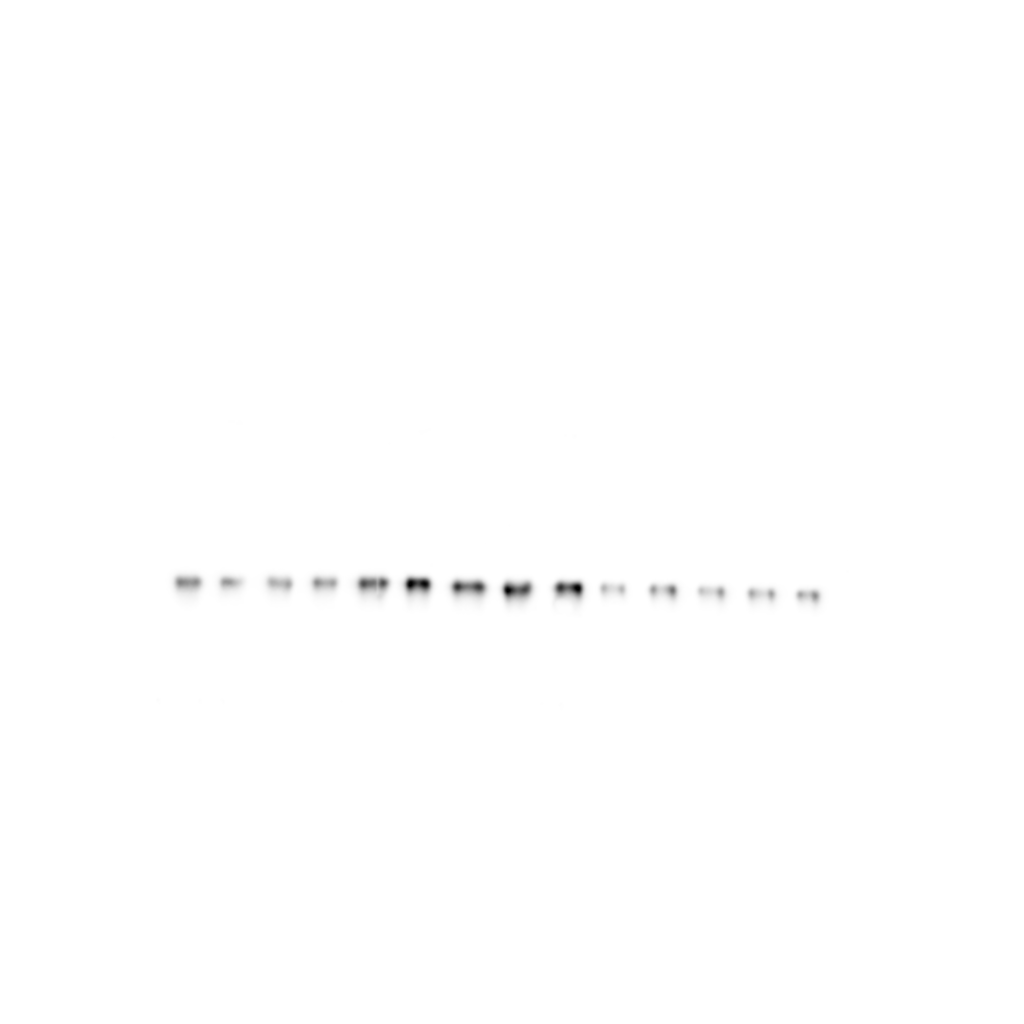


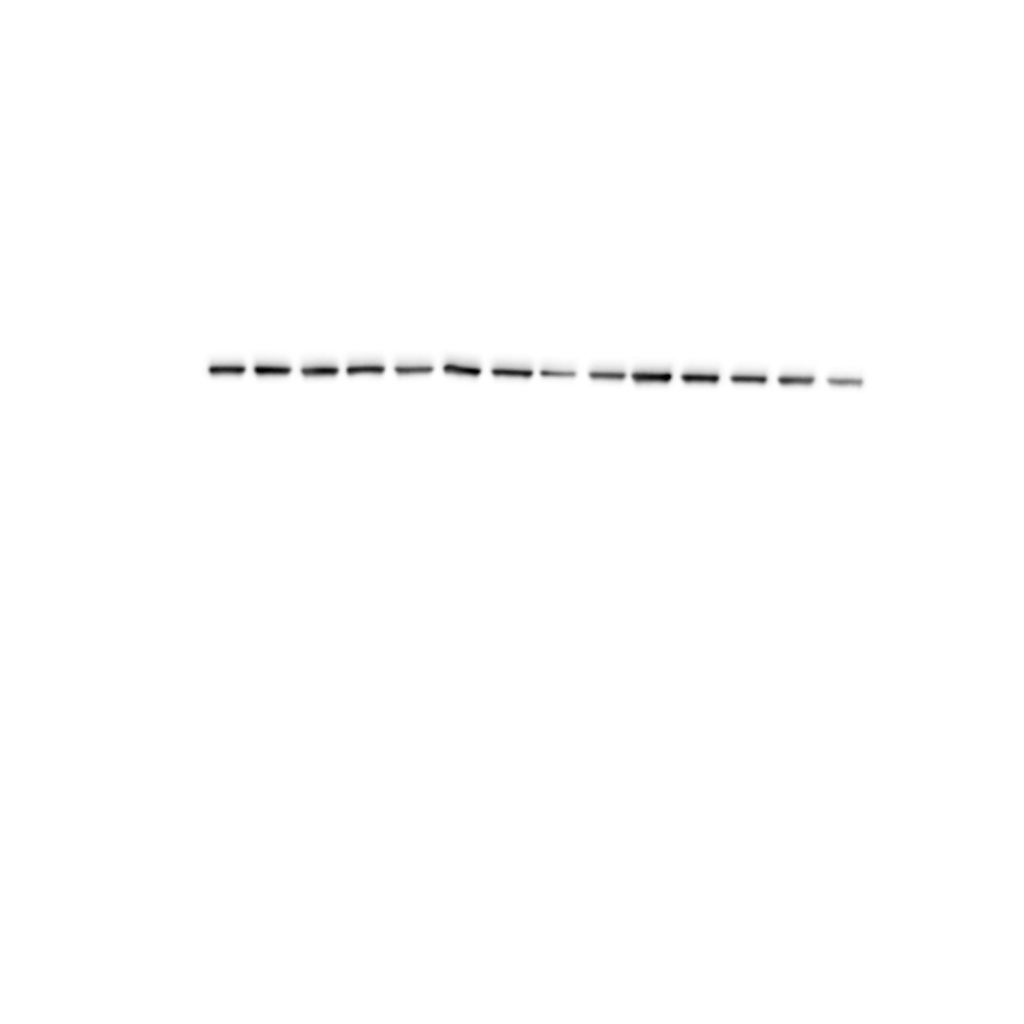

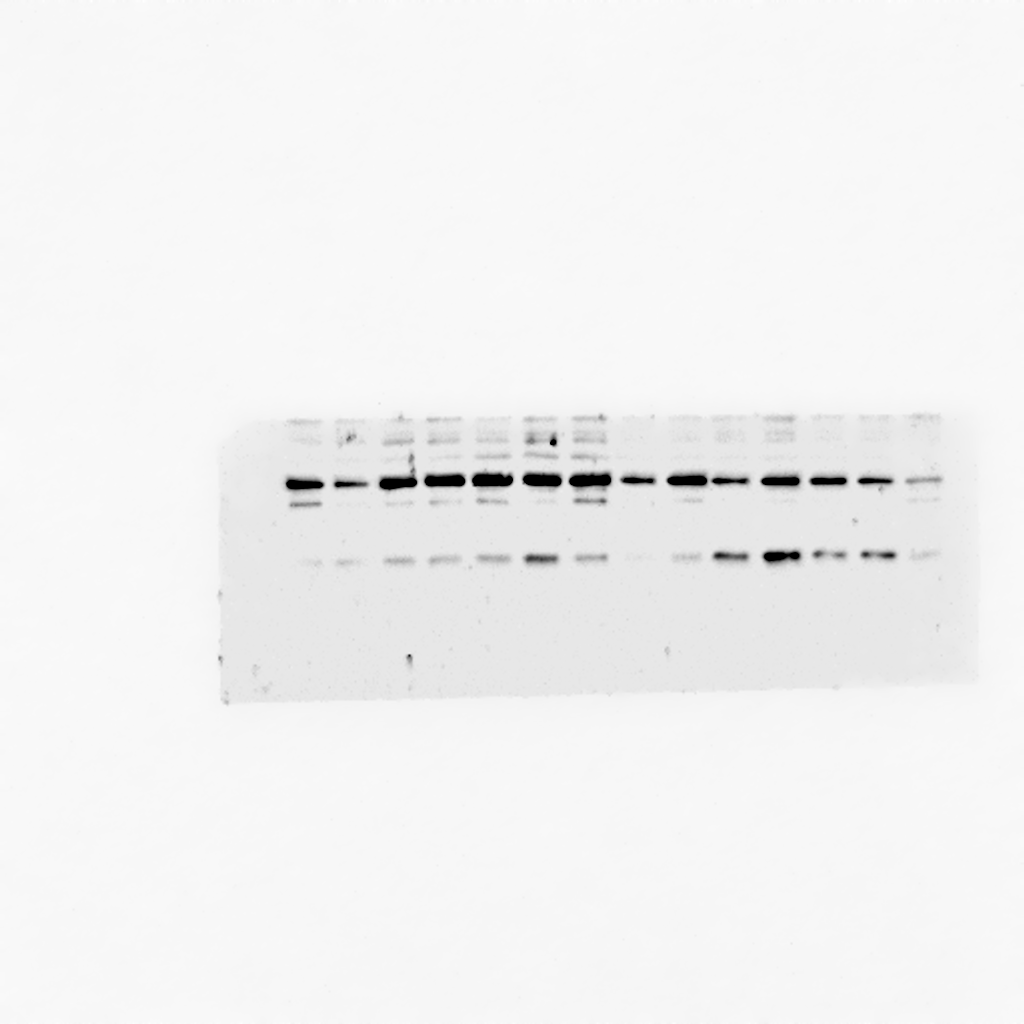


35

25

15

40

35

25

15

40

35

25

15

40

Female serum RBP4

Female liver RBP4

Female liver GAPDH

Supp Figure 2C


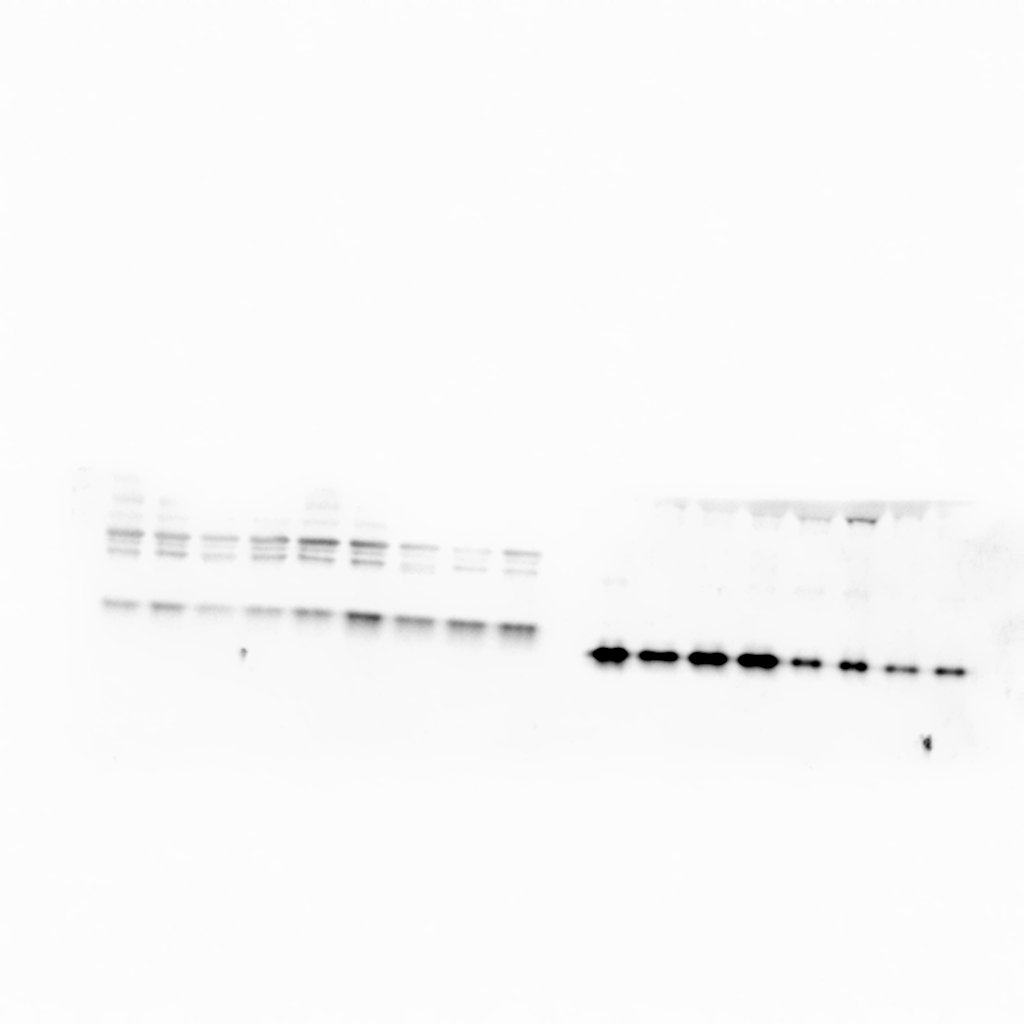

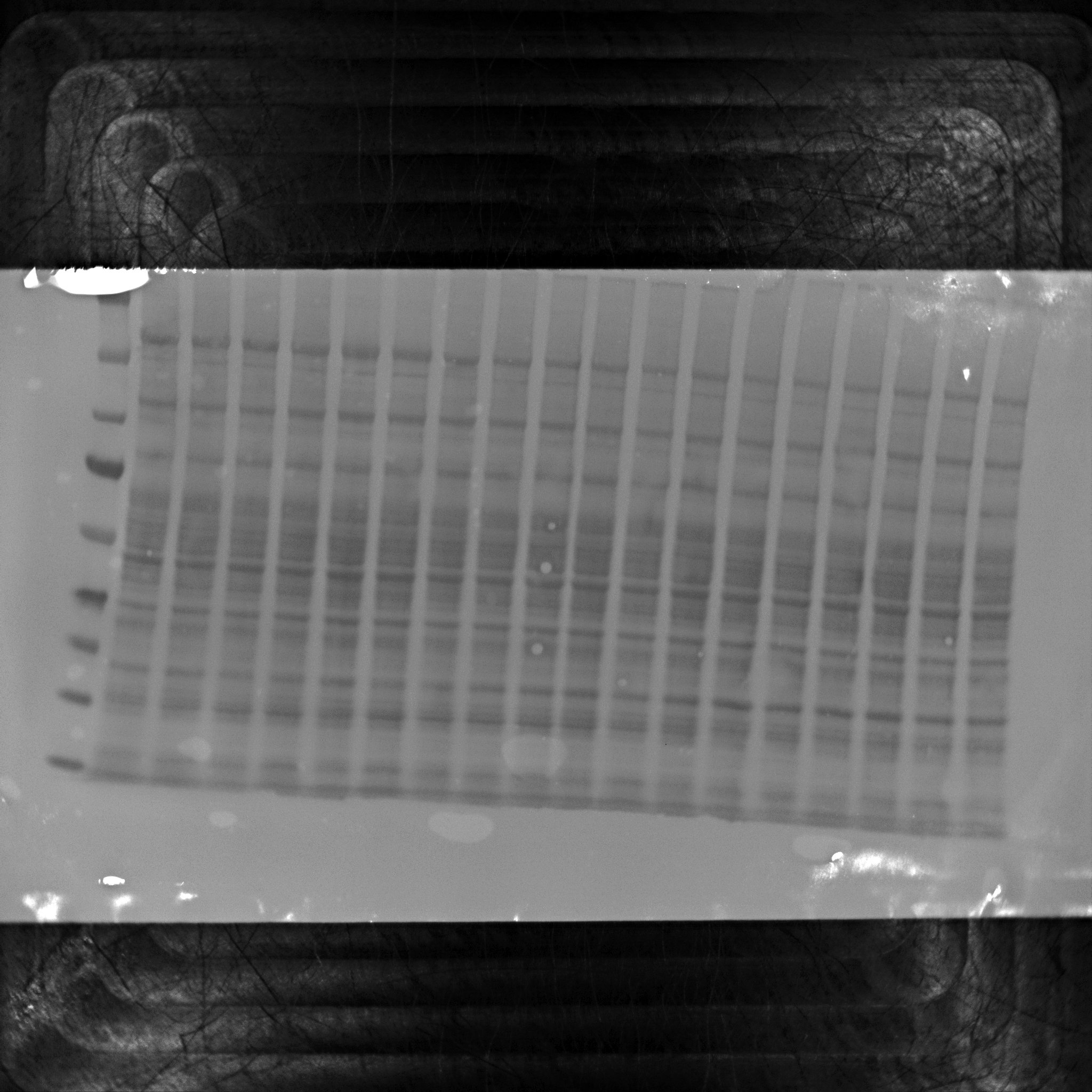


35

25

15

40

35

25

15

40

Male serum RBP4

Male liver RBP4

Supp Figure 2C

Supp Figure 4A

50

40

35

25

15

70

100

140

260

Bip

Beclin

P38/ total p38/ GAPDH

CHOP

Dashed lines indicate where membrane was cut after ponceau S Staining


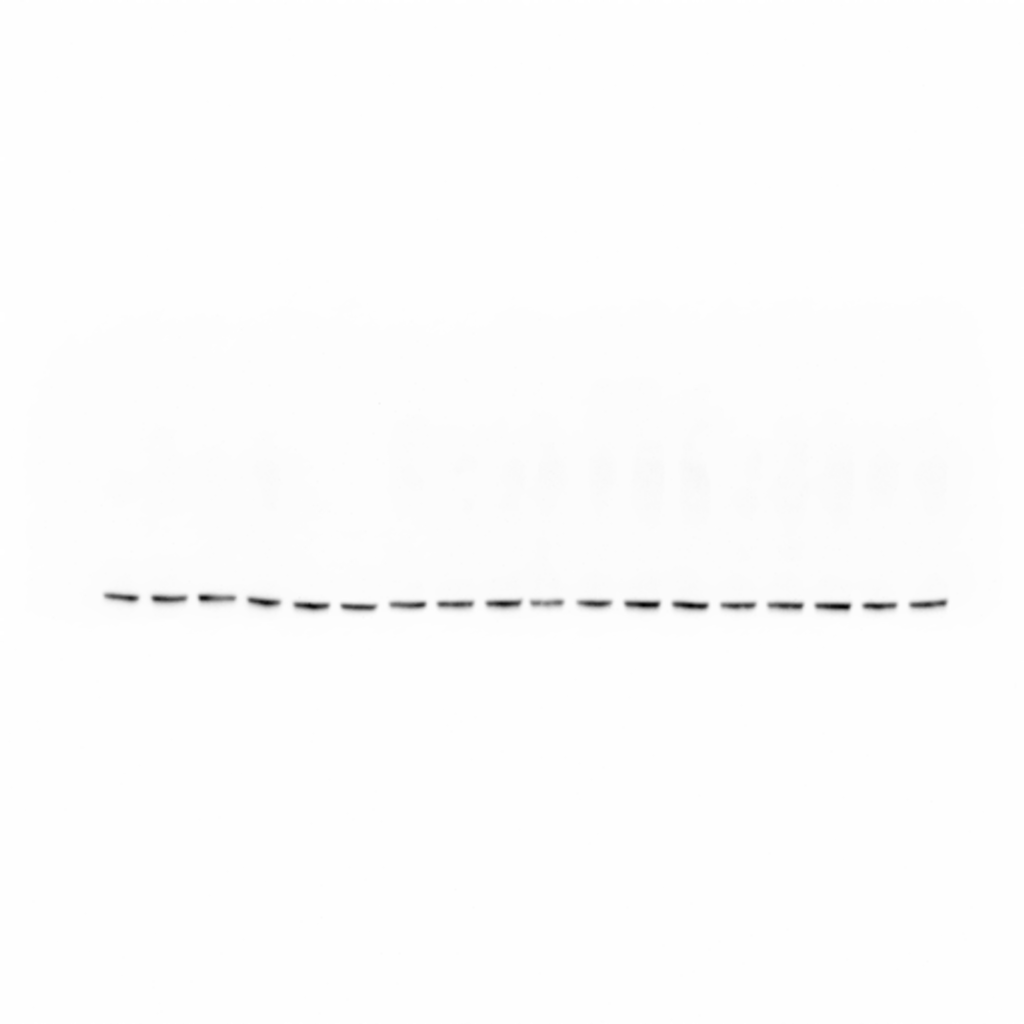

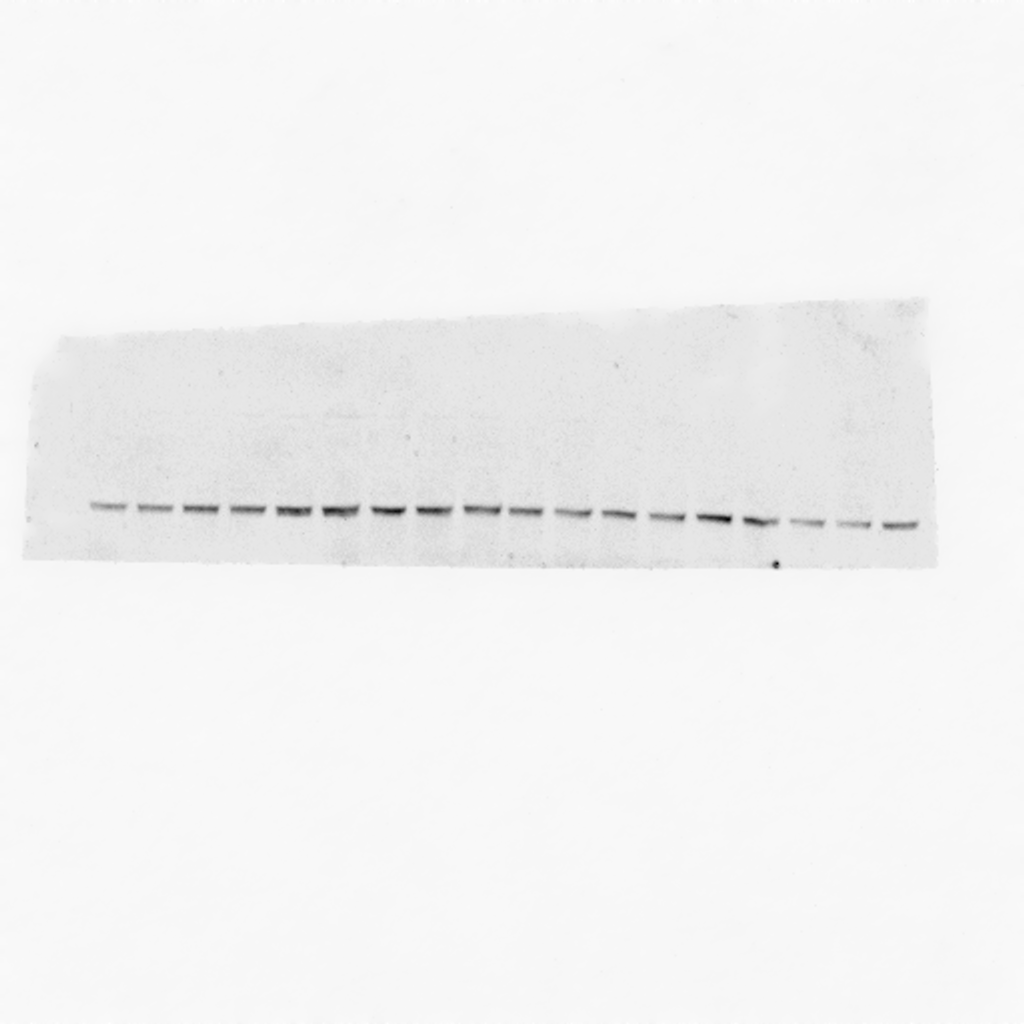

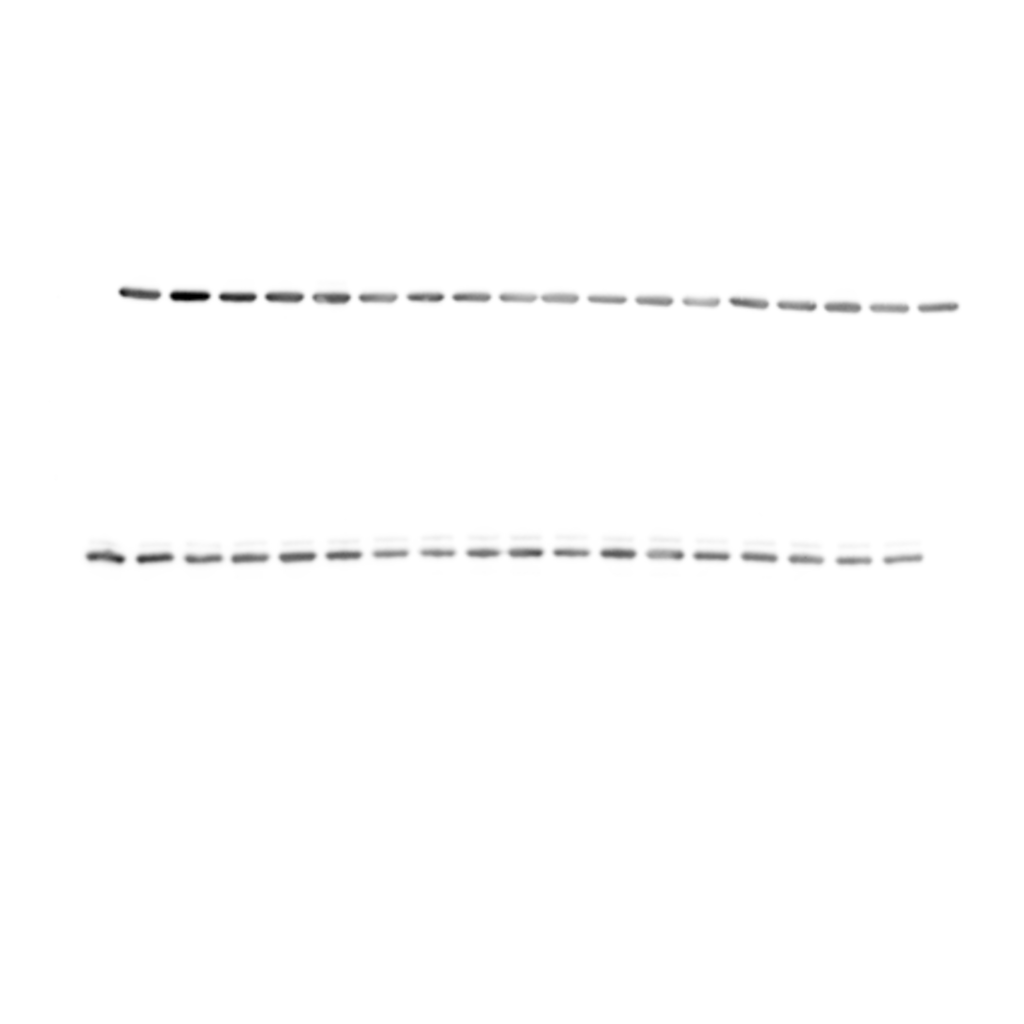


35

Supp Figure 4A

50

40

GAPDH

70

100

140

260

BiP

50

Beclin


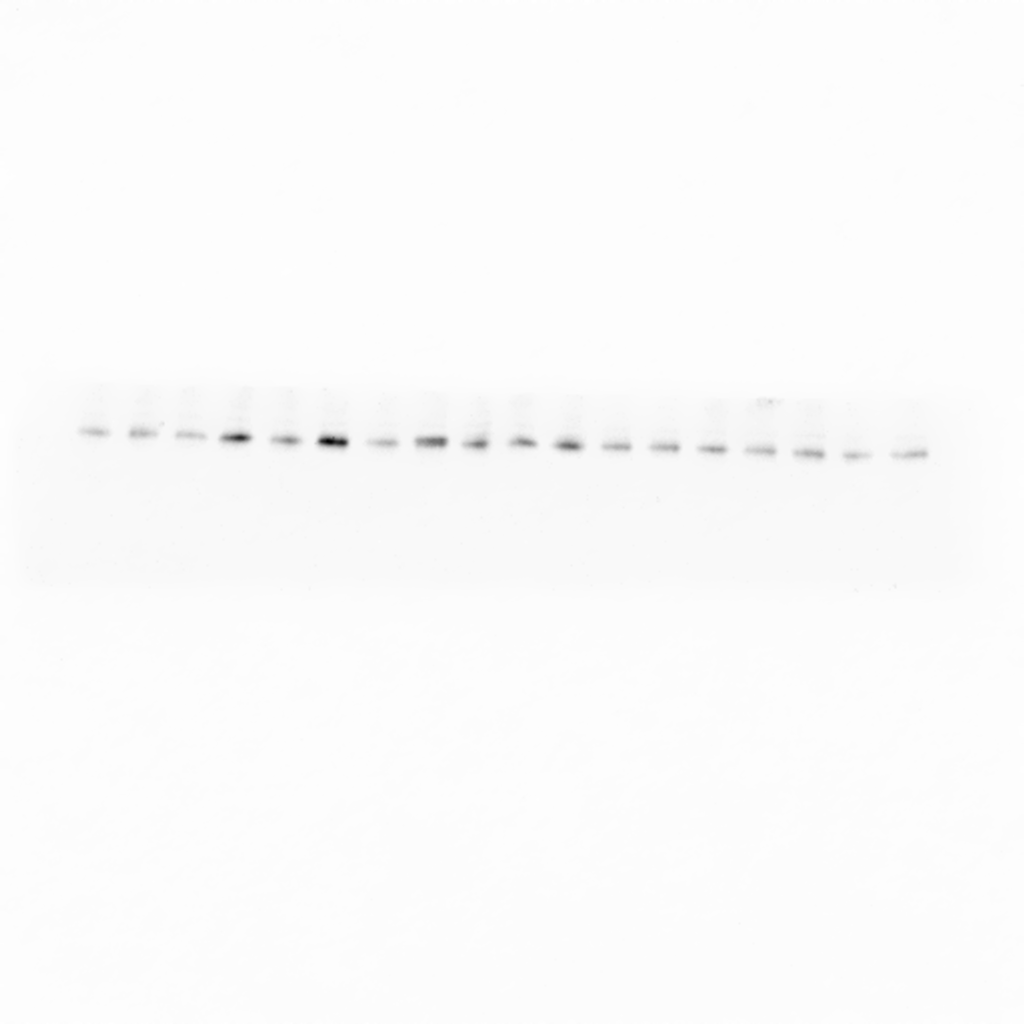

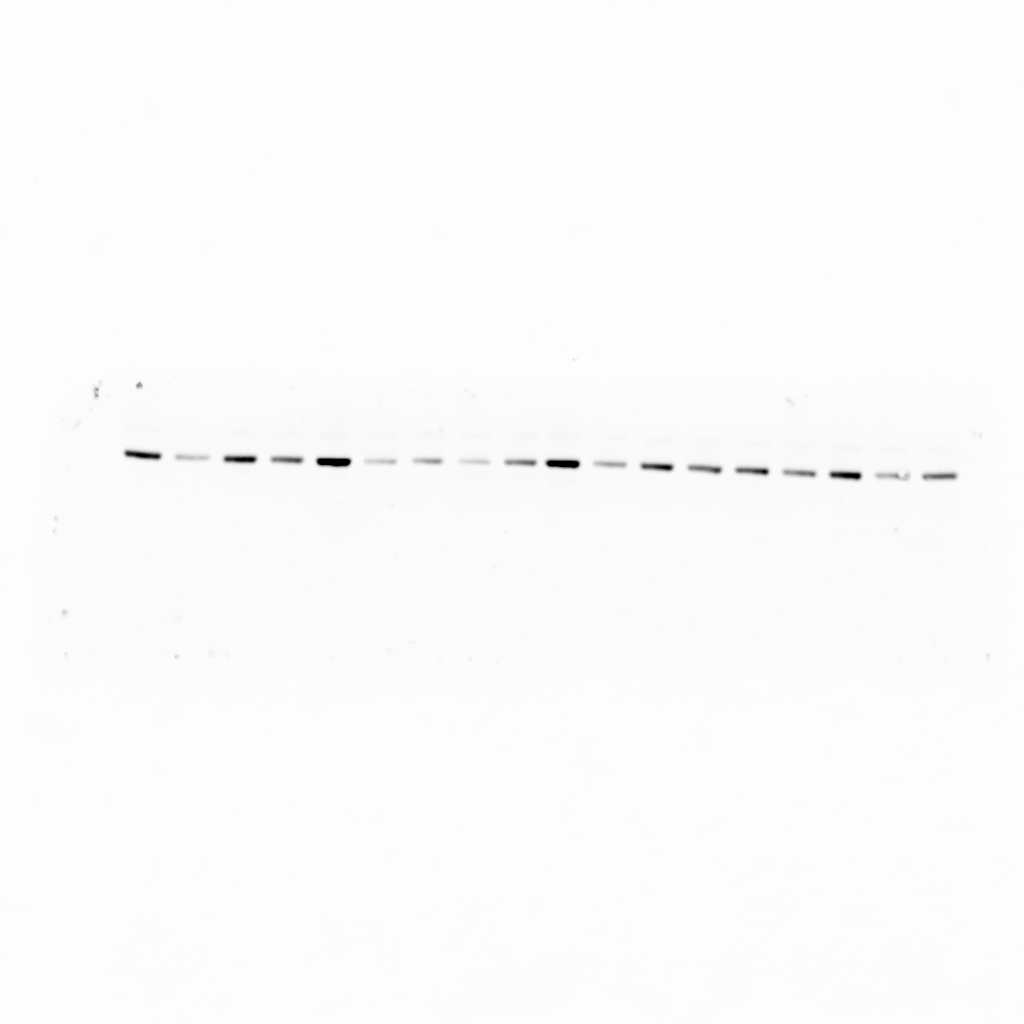

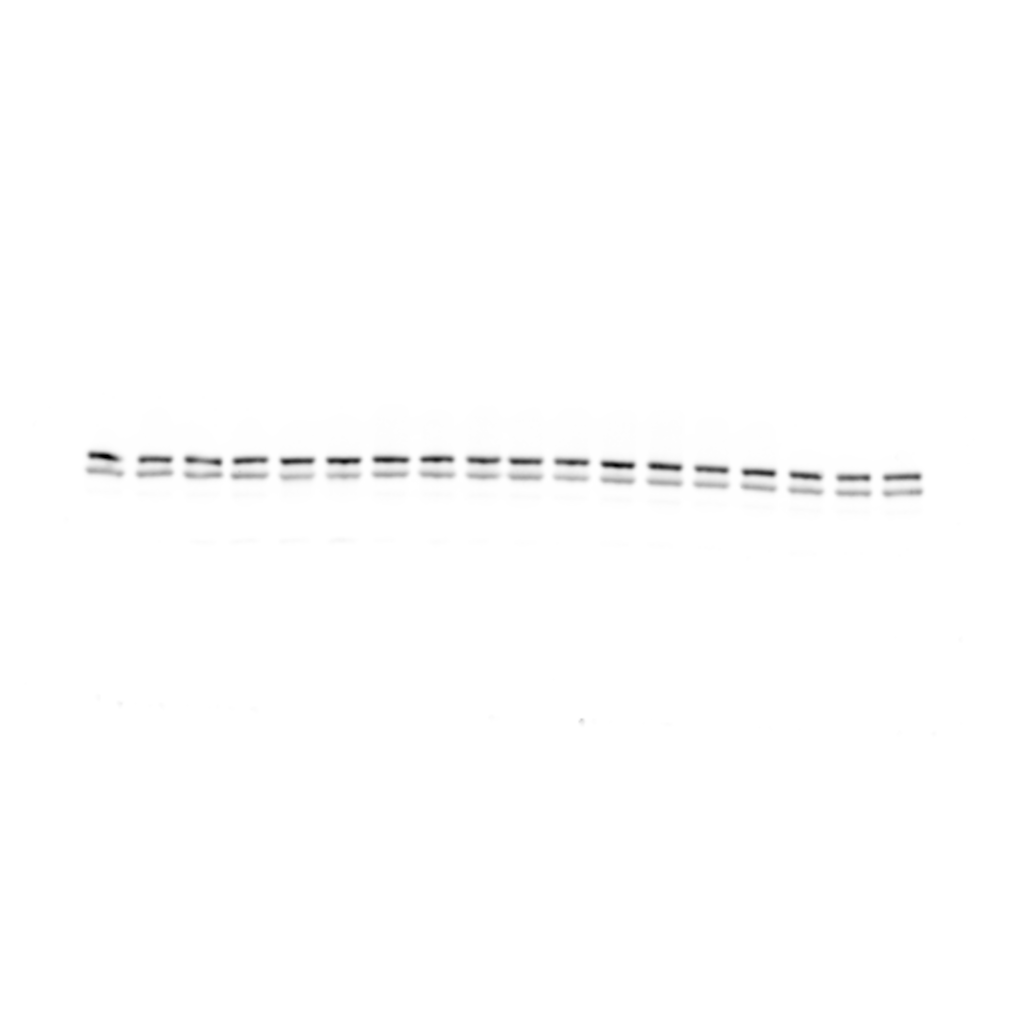


35

35

Supp Figure 4A

35

25

15

40

40

p-p38

Total p38

CHOP


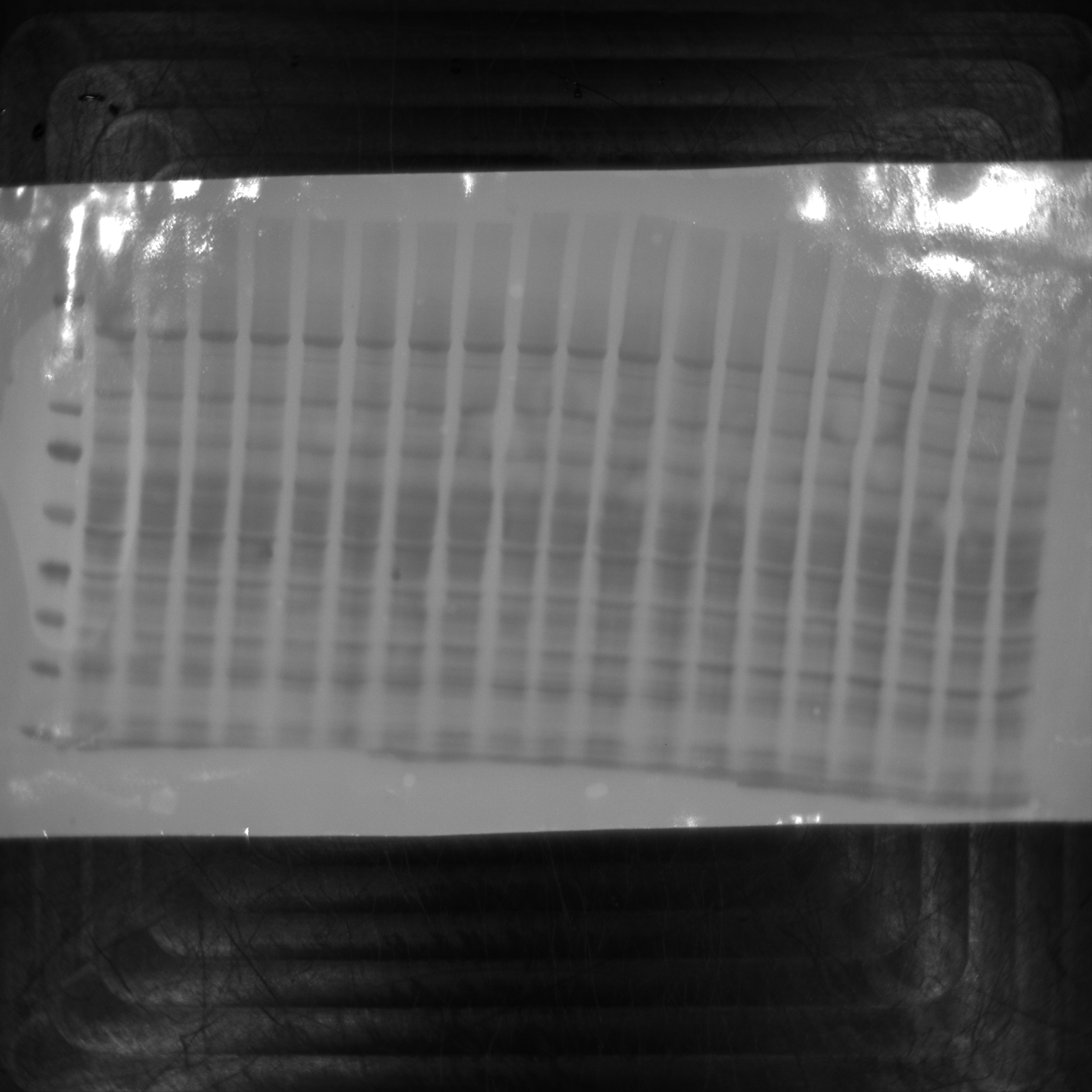


Dashed lines indicate where membrane was cut after ponceau S Staining

50

40

35

25

15

70

100

140

260

P-eif2α/total eif2α/ GAPDH

Supp Figure 4C


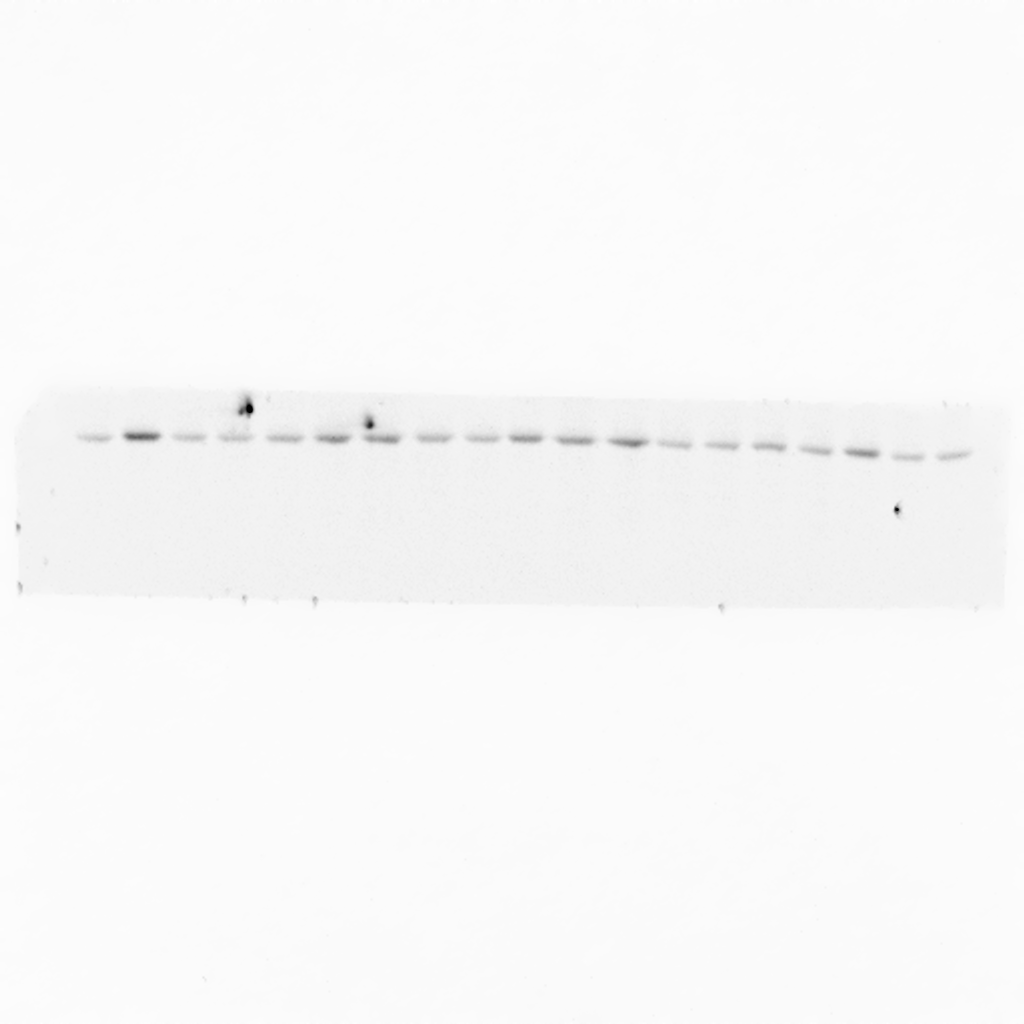

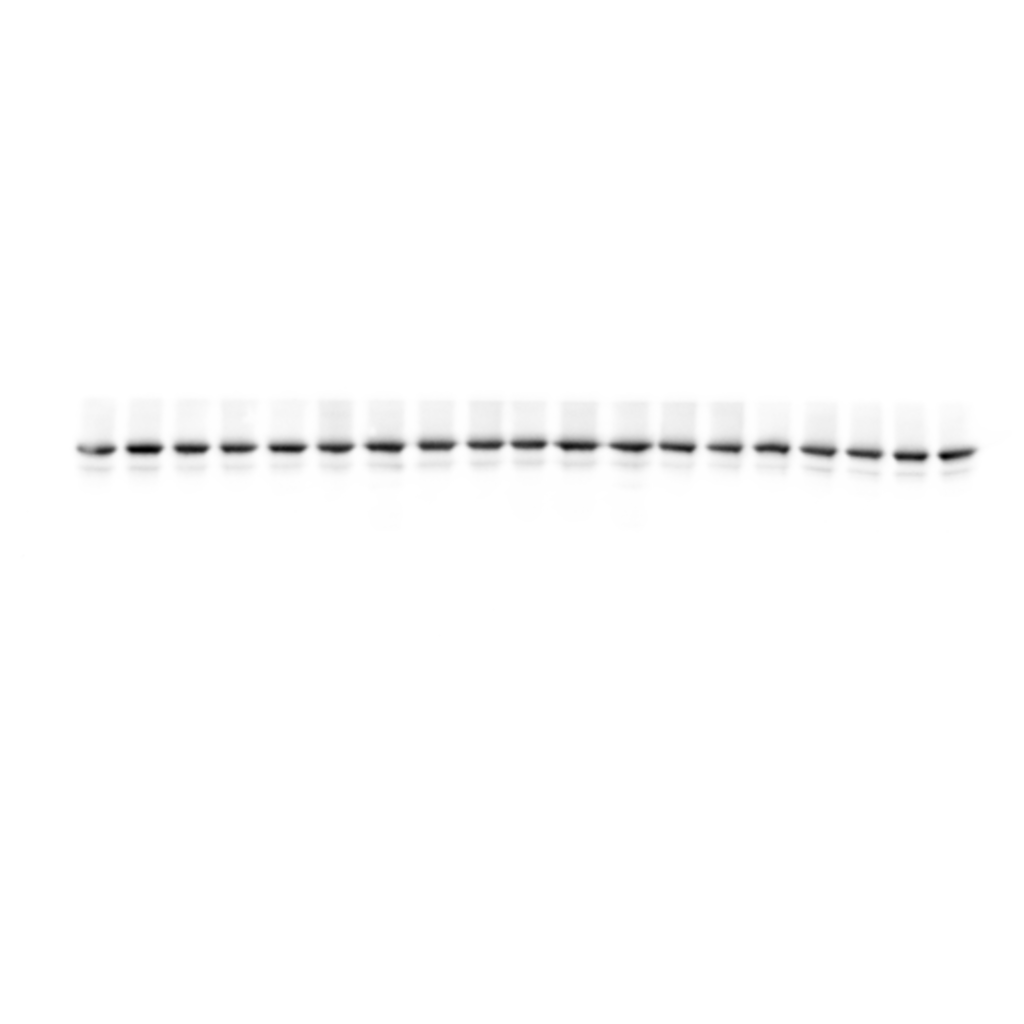


Supp Figure 4c

35

25

15

40

35

25

15

40

peif2α

Total eif2α


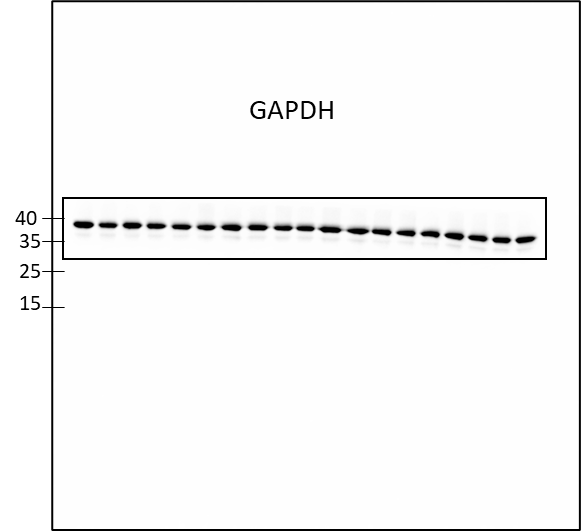

Supplement: Supplementary file 1 — Supplementary Information. [file 41598_2023_30759_MOESM1_ESM.docx]
